# Programmed cell death signatures-driven microglial transformation in Alzheimer’s disease: single-cell transcriptomics and functional validation

**DOI:** 10.3389/fimmu.2025.1610717

**Published:** 2025-07-25

**Authors:** Mi-Mi Li, Ying-Xia Yang, Ya-Li Huang, Shu-Juan Wu, Wan-Li Huang, Li-Chao Ye, Ying-Ying Xu

**Affiliations:** Department of Neurology, The Second Affiliated Hospital of Fujian Medical University, Quanzhou, Fujian, China

**Keywords:** Alzheimer’s disease, programmed cell death, microglia, single-cell, machine learning

## Abstract

**Background:**

This study aims to develop and validate a programmed cell death signature (PCDS) for predicting and classifying Alzheimer’s disease (AD) using an integrated machine learning framework. We further explore the role of S100A4 in AD pathogenesis, particularly in microglia.

**Methods:**

A total of one single-cell RNA sequencing (scRNA-seq) and four bulk RNA-seq datasets from multiple GEO datasets were analyzed. Weighted Gene Co-expression Network Analysis (WGCNA) was utilized to identify PCD-related genes. An integrated machine learning framework, combining 12 algorithms was used to construct a PCDS model. The performance of PCDS was validated using multiple independent cohorts. *In vitro* experiments using BV2 microglia were conducted to validate the role of S100A4 in AD, including siRNA transfection, Western blot, qRT-PCR, cell viability and cytotoxicity assay, flow cytometry, and immunofluorescence.

**Results:**

ScRNA-seq analysis revealed higher PCD levels in microglia from AD patients. Seventy-seven PCD-related genes were identified, with 70 genes used to construct the PCDS model. The optimal model, combining Stepglm and Random Forest, achieved an average AUC of 0.832 across five cohorts. High PCDS correlated with upregulated pathways related to inflammation and immune response, while low PCDS associated with protective pathways. *In vitro*, S100A4 knockdown in AbetaO-treated BV2 microglia improved cell viability, reduced LDH release, and partially alleviated apoptosis. S100A4 inhibition attenuated pro-inflammatory responses, as evidenced by the reduced expression of pro-inflammatory mediators (IL-6, iNOS, TNF-α) and promoted an anti-inflammatory state, indicated by increased expression of markers such as IL-10, ARG1, and YM1/2. Furthermore, S100A4 knockdown mitigated oxidative stress, restoring mitochondrial function and decreasing ROS levels.

**Conclusion:**

This study developed a robust PCDS model for AD prediction and identified S100A4 as a potential therapeutic target. The findings highlight the importance of PCD pathways in AD pathogenesis and provide new insights for early diagnosis and intervention.

## Introduction

Alzheimer’s disease (AD), a progressive neurodegenerative condition with irreversible progression, manifests through deteriorating cognitive function, episodic memory deficits, and compromised daily functioning. Representing the predominant etiology of dementia worldwide, AD constitutes approximately 60-80% of diagnosed cases (1, 2) Central to its pathogenesis are two pathological signatures: extracellular aggregates of amyloid-beta (Aβ) peptides and intraneuronal tau-based neurofibrillary pathology arising from aberrant tau phosphorylation. These pathological hallmarks instigate synaptic impairment, progressive neuronal degeneration, and cerebral volume reduction, culminating in profound behavioral alterations and mortality (3). While existing pharmacotherapies provide symptomatic relief, therapeutic standardization remains hindered by considerable clinical and neuropathological heterogeneity in AD, including divergent Aβ/tau deposition profiles and non-canonical phenotypic presentations (4). Furthermore, despite population-based studies indicating potential benefits of anti-inflammatory agents in mitigating AD risk, interventional trials have not demonstrated clinical efficacy, underscoring a persistent translational gap (5). Consequently, advancing the comprehension of AD’s molecular drivers is critical. Prioritizing the identification of novel biomarkers for preclinical detection, establishing robust prognostic stratification systems, and devising integrative frameworks to direct individualized therapeutic regimens are essential steps toward optimizing clinical management and patient outcomes.

Programmed cell death (PCD), a phylogenetically conserved process orchestrated by molecular pathways, enables controlled cellular elimination essential for developmental morphogenesis, immune regulation, and stress adaptation in both unicellular and multicellular life forms (6). To date, over 15 mechanistically distinct PCD modalities have been characterized (7). The most extensively studied types include apoptosis, a caspase-dependent, immunologically silent mechanism facilitating orderly removal of superfluous or compromised cells (8); necroptosis, a lytic, inflammation-inducing pathway regulated by RIPK1/RIPK3 signaling cascades and MLKL polymerization, culminating in cytoplasmic leakage and damage-associated molecular pattern (DAMP) dissemination (9); an inflammasome-mediated lytic process where canonical/non-canonical inflammasome pathways activate inflammatory caspases (e.g., caspase-1/4/5/11), cleaving gasdermins to form cytolytic pores and stimulate IL-1β/IL-18 secretion (10); ferroptosis, an iron-catalyzed, non-apoptotic demise marked by lipid peroxidation due to impaired redox homeostasis (GPX4 dysfunction) (11); autophagy-dependent cell death, arising from autophagic flux exceeding survival thresholds, resulting in catastrophic lysosomal degradation (12); PANoptosis, a recently defined convergent cell death modality integrating pyroptotic, apoptotic, and necroptotic machinery via multiprotein PANoptosome assemblies (13). Emerging paradigms such as cuproptosis (copper-induced proteotoxic stress) (14), paraptosis (ER/mitochondrial vacuolization) (15), and oxeiptosis (ROS-triggered, caspase-unrelated death) (16) further expand the PCD landscape. In the context of AD, concurrent dysregulation of these pathways accelerates neuropathology. Amyloid-beta pathology and tau hyperphosphorylation induce bioenergetic failure and oxidative injury, activating caspase cascades to execute apoptosis (14). Concurrently, RIPK1/MLKL-mediated necroptosis and NLRP3 inflammasome-driven pyroptosis intensify neuroinflammation through cytokine storm generation, perpetuating CNS damage (17, 18). Iron dyshomeostasis and GPX4 suppression potentiate ferroptosis, exacerbating lipid peroxidation in AD-affected neurons (19). While autophagy initially mitigates proteotoxicity, its collapse in AD promotes pathogenic protein aggregation and autophagosome-mediated degeneration (20). Notably, inter-pathway crosstalk (PANoptosis) and post-transcriptional regulatory mechanisms (RNA methylation) create feedforward loops that amplify neuronal loss and inflammatory cascades (21–23). Deciphering these interactions offers transformative insights into AD pathogenesis, highlighting PCD modulation as a promising axis for developing mechanism-based therapeutics, prognostic biomarkers, and personalized neuroprotective regimens.

The identification and validation of biomarker candidates for AD are frequently compromised by methodological inconsistencies such as cohort selection biases, intrinsic and inter-individual disease variability, assay-dependent discrepancies, and reporting biases, collectively undermining the reliability and translational applicability of findings. Consequently, systematically mapping molecular correlates of AD through multi-omic profiling is pivotal for predicting disease trajectories and informing targeted therapeutic interventions. Over the past decades, compelling evidence underscores the involvement of PCD pathways in the initiation and advancement of neurodegenerative disorders. However, current investigations lack integrative frameworks to dissect the multifaceted contributions of PCD dysregulation to AD pathobiology. Identifying reliable biomarkers is challenging due to disease heterogeneity and methodological inconsistencies. Integrated machine learning frameworks, which combine multiple algorithms, have shown great promise in analyzing complex biological data to uncover robust molecular signatures and improve prediction accuracy in various diseases (24–26). Such approaches are particularly suited for dissecting the multifaceted contributions of PCD dysregulation to AD pathobiology. To address this gap, we hypothesized that an integrative analysis combining transcriptomics with advanced machine learning could yield a robust Programmed Cell Death Signature (PCDS) capable of accurately predicting and classifying AD status. In this study, we leveraged single-cell and bulk RNA-seq data with an integrated machine learning approach to develop and validate such a PCDS. We further aimed to explore the biological role of a key gene within this signature, focusing on its function in microglia, a cell type critically involved in AD neuroinflammation. Our findings quantify interpatient variability in PCDS profiles and correlate these molecular signatures with distinct biological pathways and immune landscapes, offering a platform for patient stratification and mechanism-driven therapeutic development.

## Methods

### Data acquisition

For bulk RNA-seq analysis, the GSE132903 dataset (97 control and 98 AD brain tissues), GSE5281 dataset (74 control and 87 AD brain tissues), GSE36980 dataset (47 control and 32 AD brain tissues), GSE33000 dataset (157 control and 310 AD brain tissues), GSE106241 dataset (60 AD brain tissues), GSE48350 dataset (173 control and 80 AD brain tissues), and GSE122063 dataset (44 control and 56 AD brain tissues) were all obtained from the GEO database (https://www.ncbi.nlm.nih.gov/geo/). The GSE132903, GSE5281, GSE33000, GSE36980, and GSE48350 cohorts were utilized to construct a Programmed Cell Death Signature (PCDS), while the GSE122063 and GSE106241 datasets were selected as external validation. For scRNA-seq analysis, the GSE157827 dataset (9 control and 12 human AD brain tissues) were also downloaded from the GEO database (27). Through comprehensive literature search (28), a total of 1554 genes with 17 PCD patterns were obtained after removing duplicates (**Supplementary Table S1**). These include genes related to apoptosis (580), pyroptosis (52), ferroptosis (88), autophagy (367), necroptosis (101), cuproptosis (19), parthanatos (9), Entotic cell death (38), NETosis (32), lysosome-dependent cell death (220), alkaliptosis (7), oxeiptosis (genes), immunogenic cell death (34), anoikis (338), paraptosis (66), methuosis (8), and disulfdptosis (9).

### scRNA-seq analysis

Three files including barcodes, features, and matrix for each sample in GSE157827 dataset were downloaded to generate a Seurat object (29). Cells with low quality (<200 genes/cell, >10% mitochondrial genes, or >5% ribosomal genes) were excluded, as were genes expressed in fewer than three cells. Potential doublets were identified and removed using the *DoubletFinder* package. The gene expression matrix was normalized and scaled using the *NormalizeData* and *ScaleData* functions, respectively. Principal component analysis (PCA) was conducted on the top 2,000 most variable genes, identified via the *FindVariableFeatures* function with default parameters. Single cells were clustered in PCA space using the *FindClusters* function and visualized in two dimensions via the UMAP algorithm. In addition, Marker genes for different clusters were identified using the *FindAllMarkers* function with the default parameters: min.logfc = 0.25 and min.pct = 0.20. Clusters were manually annotated based on canonical cell markers.

### Bulk dataset preprocessing and analysis

RNA sequencing data processing commenced with logarithmic transformation of the raw count matrix, followed by inter-sample normalization using the *normalizeBetweenArrays* algorithm from the limma R package (30). To address technical confounding factors across heterogeneous sample batches, cross-platform harmonization was implemented via the *Combat* function in the sva library (31). Data integration quality was subsequently verified through principal component analysis (PCA), which visualized inter-group variance attributable to residual batch effects. Subsequently, differentially expressed genes were determined using the limma package.

### Weighted gene co-expression network analysis

WGCNA is an algorithm designed to identify genetic interactions in a weighted manner (32). It constructs a gene co-expression network module that closely correlates with clinical traits through systematic biological methods. The analysis was conducted using the WGCNA R package. In summary, the top 25% of highly variable genes (7932 genes) were selected as input genes. The input samples were filtered using the *goodSamplesGenes* function and were clustered to detect outliers. When the scale-free fit R² approaches 0.9, a soft-thresholding power of 10 and utilized to build a scale-free network, which was subsequently converted into a topological overlap matrix (TOM). Subsequently, the dynamic hybrid cleavage method was employed to identify multiple gene modules based on TOM-derived dissimilarity (minModuleSize =200), and the *mergeCloseModules* function was applied to merge similar modules. Finally, gene significance (GS) for AD, module members (MM) within the modules associated with AD, and the pearson correlations between GS and MM were determined.

### Construction of PCDS based on an integrated machine learning−based framework

To develop robust PCDS, we evaluated 12 machine learning (ML) approaches. These methods were systematically integrated into a computational framework leveraging intersecting transcriptional profiles associated with PCD across five independent AD cohorts (GSE132903, GSE33000, GSE36980, GSE48350, GSE5281). A total of 134 model permutations, including spanning penalized regressions (Lasso, Ridge, ElasticNet), ensemble learners (Random Forest, Gradient Boosting Machines, XGBoost), and probabilistic classifiers (Naive Bayes), were incorporated. A dual-algorithm approach, including separating feature selection from prediction rule derivation, was implemented to mitigate overfitting. Nested cross-validation ensured rigorous model training, with each permutation generating classification models. Predictive performance was quantified via receiver operating characteristic (ROC) curve analysis across cohorts, followed by hierarchical clustering heatmaps for comparative visualization of area-under-curve (AUC) metrics. The model demonstrating superior mean AUC values was designated optimal, with its feature subset prioritized for downstream mechanistic investigation.

### Calculation of PCDS score

The PCDS score model was established using the following formula:


$$PCDS\;score=\sum_{i=1}^{9}\text{βi}\times \text{Ei}$$

Where i denotes individual PCD modules identified via ML frameworks, βi indicates module-specific regression coefficients, and Ei represents normalized expression levels. Patients were stratified into high/low PCD-AS subgroups using cohort median thresholds.

### Prognostic nomogram development

A multivariable nomogram integrating PCDS and clinical variables (age, sex) was constructed using the rms R package. Predictive scores for individual covariates were summated to estimate total AD risk. Model accuracy was validated via calibration plots comparing predicted versus observed outcomes, supplemented by decision curve analysis (DCA) to quantify clinical utility.

### Pathway enrichment profiling

Gene Set Variation Analysis (GSVA) is an unsupervised method for evaluating the enrichment of predefined gene sets in transcriptomic data, allowing for the inference of biological functions within samples (33). In this study, GSVA was implemented to evaluate predefined molecular pathway activities (hallmark gene sets from MSigDB database). Pathways with absolute GSVA t-scores ≥2 were considered differentially regulated. Additionally, differentially enriched pathways between groups were analyzed using the GSEA package (34). For this analysis, the c2.cp.kegg.v7.1.symbols.gmt gene set was used as a reference, and false discovery rate (FDR) <0.05 was established.

### Immune infiltration analysis

Immune cell infiltration patterns were deconvolved using the IOBR R package (35), integrating five computational methods: Cell-type Identification by Estimating Relative Subsets of RNA Transcripts (CIBERSORT), Microenvironment Cell Populations-counter (MCPCounter), xCell, EPIC, and QUANTISEQ. Each approach provided distinct insights into gene expression profiles and immune cell abundance, collectively offering a comprehensive understanding of the AD immune landscape. Additionally, immune checkpoint molecules, including spanning antigen presentation, costimulatory/coinhibitory receptors, and cytokine signaling axes, were analyzed to delineate lymphocyte activation levels across PCDS subgroups.

### Cell–cell communication

The CellChat package was utilized to integrate gene expression data and analyze differences in cell–cell communication between groups (36). Briefly, we generated a normalized expression matrix across distinct cell types to create a CellChat object. The default CellChatDB was selected as the ligand-receptor database. We identified overexpressed ligands or receptors in cell groups using the *identifyOverExpressedGenes* function with default parameters and inferred overexpressed ligand-receptor interactions using the *identifyOverExpressedInteraction* function. Gene expression data were projected using the *projectData* function. Communication probability and potential communication networks were calculated using the *computeCommunProb* function. Cell-cell communication networks were aggregated using the *aggregateNet* function. Finally, the *netAnalysis_computeCentrality* function was applied to determine the signaling effects and crucial contribution signals of cell types between distinct groups.

### Trajectory analysis

The Monocle2 R package was employed to conduct pseudotime analysis, enabling the computation and ordering of gene expression changes in cells collected from different groups using an unsupervised approach (37). To extract the cell clusters of interest, the *subset* command from the Seurat package was utilized. Subsequently, a Monocle object was created using the *newCellDataSet* function from the Monocle2 package with *lowerDetectionLimit* set to 0.5. Genes exhibiting low expression levels were filtered using the *detectGenes* function, with a minimum expression threshold of 0.1. The *differentialGeneTest* function identified differentially expressed genes along the trajectory with a q-value threshold of 0.01. Dimensionality reduction was performed using the DDRTree method, after which the *orderCells* function ordered the cells based on their expression profiles. Finally, the *plot_cell_trajectory* and *plot_pseudotime_heatmap* functions were used for visualization following cell ordering.

### Cell culture

This study received approval from the Institutional Animal Care and Use Committee at The Second Affiliated Hospital of Fujian Medical University. The BV2 murine microglial cell line and HT-22 hippocampal neuronal cell line were acquired from Procell Life Science & Technology Co., Ltd. (Wuhan, China). Cells were maintained in Dulbecco’s Modified Eagle Medium (DMEM; Gibco, USA) supplemented with 10% fetal bovine serum (FBS), 50 U/mL penicillin, and 50 µg/mL streptomycin, and incubated at 37°C in a humidified atmosphere containing 5% CO_2_. For subculturing, adherent cells were detached using 0.25% trypsin-EDTA solution (Gibco, USA) upon attaining 80% confluency.

### Amyloid beta oligomers treatments in BV2 cells

Briefly, a total of 1 mg of AbetaO powder obtained from ChinaPeptides Co., Ltd (Shanghai, China) was dissolved in DMSO to prepare a stock solution with a concentration of 1 mM, followed by the dilution with DMEM to a final concentration of 5 μM. The soluble fraction was stored at -80°C. BV2 cells were subsequently incubated with 5 μM AbetaO for 24 hours at 37°C to establish an Alzheimer’s disease (AD) cell model.

### Small interfering RNA and transfection

Scrambled siRNA (si-NC) or S100A4 siRNA (si-S100A4) (RiboBio, Guangzhou, China) was transfected into BV2 cells utilizing the Lipofectamine 2000 transfection reagent (Invitrogen, CA, USA). Briefly, an equal volume of each siRNA solution was combined with Lipofectamine 2000, gently mixed, and incubated for 20 minutes. BV2 cells at 70-80% confluency in 6-well plates were then transfected with the mixture in OptiMEM (Invitrogen, Carlsbad, CA, USA) for 24 hours. Following transfection, cells were cultured in antibiotic-free medium for an additional 72 hours before proceeding with further experiments.

### Western-blot analysis

BV2 microglial cells were collected and lysed in ice-cold RIPA lysis buffer (Beyotime, China) supplemented with protease/phosphatase inhibitors and PMSF. The lysate was centrifuged at 12000 rpm for 30 minutes at 4°C, after which the clarified fraction was isolated. Protein concentrations were determined using a bicinchoninic acid (BCA) assay kit (Thermo Fisher, USA). Equal protein aliquots from each group were resolved on 10% SDS-PAGE gels and electroblotted onto PVDF membranes (Millipore, USA). Membranes were blocked with 5% bovine serum albumin (BSA) in Tris-buffered saline containing 0.1% Tween-20 (TBST) for 1 hour at room temperature, followed by overnight incubation at 4°C with a primary antibody targeting S100A4 (1:10000; Proteintech, USA). After TBST washes, membranes were incubated with horseradish peroxidase (HRP)-conjugated secondary antibodies. Immunoreactive bands were detected using a chemiluminescent substrate (Bio-Rad, USA), and densitometric quantification was performed with ImageJ software.

### Quantitative real-time PCR

Total RNA was extracted from BV2 microglial cells using TRIzol reagent (Invitrogen, CA, USA) according to the manufacturer’s protocol. The concentration and integrity of RNA were evaluated using a NanoDrop 2000 spectrophotometer (Thermo Fisher Scientific, USA). Complementary DNA (cDNA) was synthesized using the RevertAid First Strand cDNA Synthesis Kit (Thermo Fisher Scientific, USA), and qRT-PCR was performed on a 7500 Real-Time PCR System (Applied Biosystems, USA) with specific primer sequences listed in **Supplementary Table S2**. Relative mRNA expression levels were calculated using the 2^-ΔΔCt^ method and normalized to β-actin. Data are presented as fold changes relative to the control.

### Cell viability assay

The viability of BV-2 cells was assessed using the Cell Counting Kit-8 (CCK-8) (Beyotime, China). Briefly, BV-2 cells were seeded into 96-well plates at a density of 5000 cells per well. After incubation for 24 hours to allow cell attachment, 10 μL of CCK-8 reagent was added to each well, and the plates were incubated for an additional 2 hours at 37°C. The optical density (OD) at 450 nm was then measured using a microplate reader to determine cell viability.

### Cytotoxicity assay

The cytotoxicity of BV2 cell line was assessed by a lactate dehydrogenase (LDH) assay kit (Beyotime, China). Briefly, when BV2 cells in 96-well plates reached the desired confluence, the supernatants were collected and incubated with the LDH detection reagent for 2 hours at 37°C. Absorbance was measured at 490 nm using a microplate reader. The percentage of LDH release was calculated using the formula: LDH release (%) = supernatant LDH release/total LDH release x 100.

### Flow cytometry

Apoptosis of BV2 microglia was assessed using an Annexin V-FITC/Propidium Iodide (PI) Apoptosis Detection Kit (BD Biosciences, USA) according to the manufacturer’s instructions with minor modifications. BV2 cells were collected by trypsinization, rinsed with ice-cold PBS, and brought to a concentration of 1×10^6^cells/mL in 100 μL of 1X Annexin Binding Buffer. The cell suspension was then co-incubated with 5 μL of Annexin V-FITC and 5 μL of PI. After brief vortexing and a 15-minute incubation at 37°C shielded from light, an additional 400 μL of binding buffer was introduced. Flow cytometric data acquisition was performed within an hour on a BD flow cytometer and analyzed using FlowJo software.

Terminal deoxy nucleotide transferase-mediated nick-end labeling (TUNEL) assay Neuronal apoptosis in treated HT-22 cells was quantified using the *In Situ* Cell Death Detection Kit (Roche, USA) according to manufacturer’s instructions. In brief, cell cultured on coverlips was fixed with 4% paraformaldehyde for 15 min and subsequently permeabilized with permeabilized with 0.1% Triton X-100 in 0.1% sodium citrate for 3 min on ice. Cells were then incubated with the TUNEL reaction mixture, containing terminal deoxynucleotidyl transferase (TdT) and a fluorescently labeled dUTP for 1h at 37°C in the datk. Images were acquired using a fluorescence microscope. The ratio of TUNEL-positive cells relative to the total number of DAPI positive cells was determined.

### Reactive oxygen species measurement

The levels of intracellular ROS were quantified by a 2’,7’-dichlorofluorescein diacetate (DCFH-DA, Sigma-Aldrich, USA) immunofluorescence probe following the manufacturer’s instructions. Briefly, BV2 cells from each treatment group were incubated with 10 μM DCFH-DA for 30 minutes in the dark. Following incubation, the cells were washed with phosphate-buffered saline (PBS) and visualized under a fluorescence microscope (Olympus, Japan). The DCFH-DA fluorescence intensity was quantified using ImageJ software.

### mitochondrial membrane potential detection

MMP was assessed using a JC-1 fluorescent probe according to standardized protocols. BV2 microglial cells plated in 12-well culture dishes underwent designated treatments before exposure to JC-1 working solution (5 μM) for 20 minutes at 37°C under light-protected conditions. Elevated MMP facilitates JC-1 aggregation within mitochondria, emitting red fluorescence, whereas diminished MMP results in cytoplasmic monomeric JC-1 displaying green fluorescence. Post-staining, cells were rinsed with PBS, and fluorescence signals were quantified via fluorescence microscope (excitation 488 nm, emission 594 nm).

### Immunofluorescence

For Immunofluorescence, treated BV2 cells on coverslips were fixed in 4% paraformaldehyde, permeabilized with 0.25% Triton X-100 for 10 minutes, and blocked with 3% bovine serum albumin (BSA) in PBS-Tween (0.05%) for 1 hour. Next, coverslips were incubated overnight at 4°C with the following primary antibodies: rabbit anti-CD86 (1:100; Abclonal, China), rabbit anti-CD206 (1:100; Abclonal, China), mouse anti-IBA1 (1:200; Abclonal, China). After PBS washes, cells were incubated with Alexa Fluor 488/594-conjugated secondary antibodies (Invitrogen, USA) for 2 hours in darkness, counterstained with DAPI, and imaged using a fluorescence microscopy system. The average fluorescence intensity was measured using the ImageJ software.

### Statistical analyses

Statistical analyses were conducted using GraphPad Prism 8.0 (GraphPad Software, USA). Quantitative results of experimental data are expressed as mean ± standard deviation (SD), with group sample sizes detailed in corresponding figure legends. For comparisons involving two cohorts, significance was determined via two-tailed Student’s t-test. Multi-group comparisons employed one-way analysis of variance (ANOVA), with Bonferroni-adjusted pairwise analyses applied *post hoc* to mitigate type I error inflation. A probability threshold of p < 0.05 defined statistical significance.

## Results

### Distinct cell-specific distribution of PCD patterns in AD based on scRNA-seq atlas

**Figure 1:**
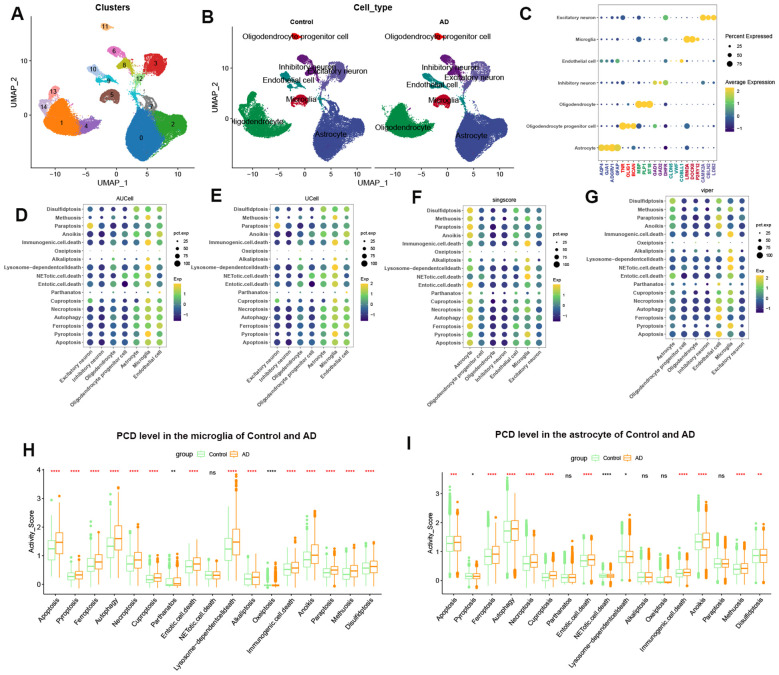
Cell-type-specific PCD dynamics in AD pathogenesis. **(A)** Uniform Manifold Approximation and Projection (UMAP) visualization of 44,120 single-cell transcriptomes following unsupervised clustering. **(B)** UMAP plots showing 44,120 cells with type annotation (astrocyte, microglia, excitatory neuron, inhibitory neuron, endothelial cell, oligodendrocyte, and oligodendrocyte progenitor cell). **(C)** Heatmap depicting cell type-specific marker gene expression. **(D–G)** Multivariate score distribution bubble plot evaluating PCD activation across diverse cell types via multiple computational metrics. **(H, I)** Comparative distribution analyses of PCD pathway enrichment in astrocyte and microglia between AD and controls. Significance thresholds: ^****^ *p*<0.0001, ^***^ *p*<0.001, ^**^ *p*<0.01, ^*^ *p*<0.05, ns *p*>0.05.

To estimate the cell-specific expression of programmed cell death (PCD) patterns in AD, we first performed scRNA-seq analysis on the GSE157827 dataset. Cells from 10 control individuals and 11 AD patients were clustered into 15 distinct populations using the UMAP algorithm (**Figure 1A**). These clusters were annotated into 7 main cell types based on known marker genes, including astrocytes, microglia, excitatory neurons, inhibitory neurons, endothelial cells, oligodendrocytes, and oligodendrocyte progenitor cells (**Figure 1B**). Distinct cellular markers within these classifications were visualized via a bubble plot (**Figure 1C**). We then evaluated the distribution and intensity of 17 PCD scores across different cell types using four common algorithms: AUCell, UCell, singscore, and viper (**Figures 1D–G**). Our analysis revealed that astrocytes exhibited higher levels of disulfidptosis, while microglia showed the highest levels of immunogenic cell death, alkaliptosis, lysosome-dependent cell death, NETotic cell death, entotic cell death, and cuproptosis. Endothelial cells demonstrated elevated levels of anoikis, parthanatos, and ferroptosis. Moreover, most PCD pattern scores in astrocytes and microglia were consistently higher in AD cortical tissue compared to normal tissue, with the exception of pyroptosis, parthanatos, alkaliptosis, oxeiptosis, and paraptosis (**Figures 1H, I**). These findings suggest that altered PCD activity may significantly contribute to AD progression.

### Identification of pivotal PCD via comprehensive screening

**Figure 2:**
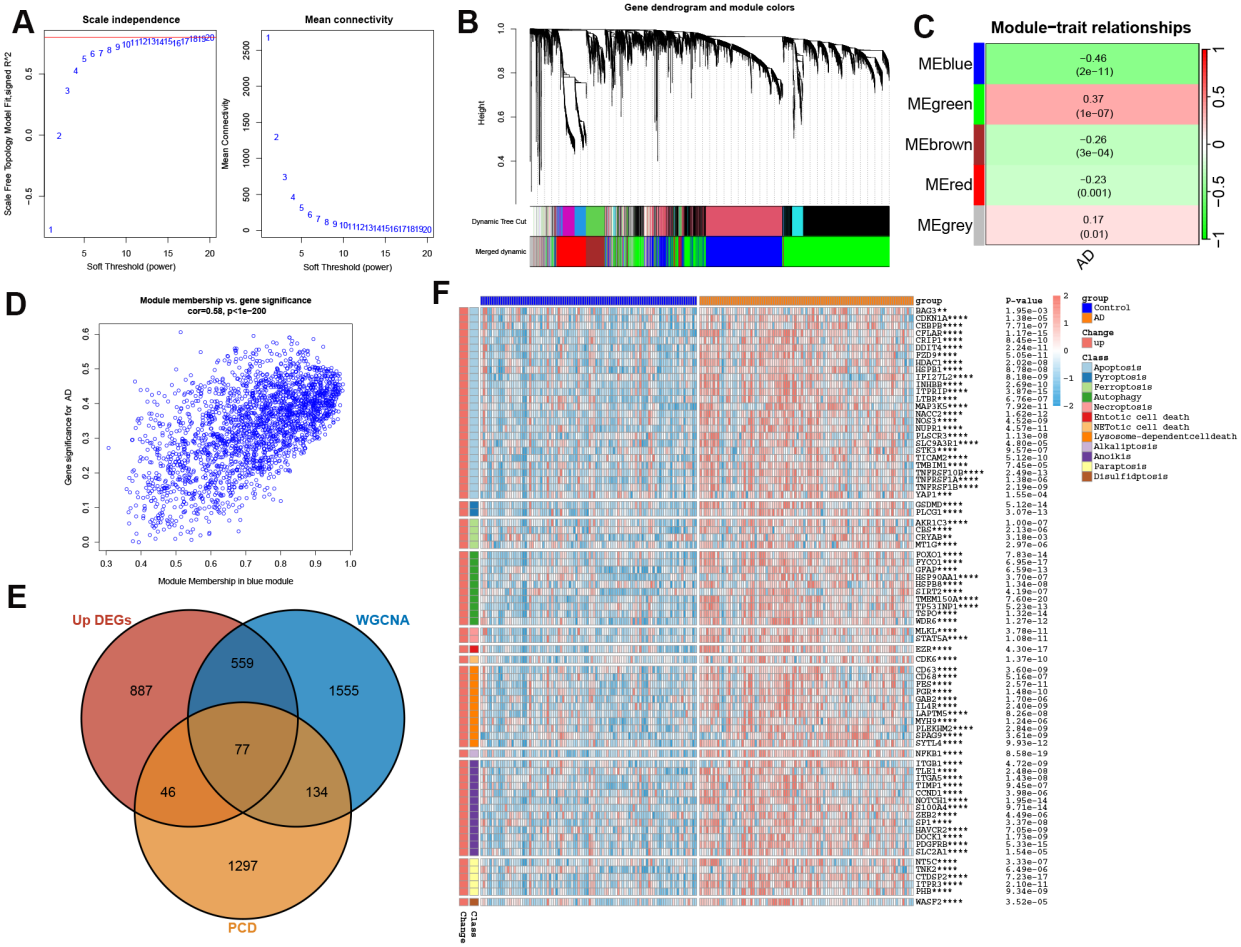
Identification of pivotal PCD-related genes. **(A)** Weighted Gene Co-expression Network Analysis (WGCNA) power threshold selection via scale-free topology metrics. **(B)** Hierarchical clustering dendrogram of co-expressed gene modules. **(C)** Module-trait correlation matrix linking gene clusters to clinical phenotypes. **(D)** Scatterplot correlating intramodular connectivity (kME) with AD association strength (GS) for the hub-enriched blue module. **(E)** Consensus PCD drivers identified through integrative analysis of differential expression (DEGs), WGCNA modules, and curated PCD-associated genes. **(F)** Heatmap showing the expression profiles of the identified 77 PCD-related genes

We initially investigated the differences between AD and control groups based on the GSE132903 dataset, identifying 3115 differentially expressed genes (DEGs), of which 1569 were up-regulated and 1546 were down-regulated (**Supplementary Table S3**). Subsequently, we performed WGCNA to identify modules associated with AD. A soft threshold power of 10 was selected, yielding a scale-free index of 0.9 and favorable mean connectivity (**Figure 2A**). This resulted in the formation of five gene modules (**Figure 2B**, **Supplementary Table S4**). The MEblue module was significantly correlated with AD (R = -0.46, p < 0.0001) (**Figure 2C**), with a correlation coefficient between gene significance and module membership of 0.58 (**Figure 2D**). In addition, we further intersected 1569 up-regulated genes from AD samples, 2325 genes from the MEblue module, and 1554 genes encompassing 17 PCD patterns, identifying 77 overlapping genes (**Figure 2E**). Afterward, we demonstrated the expression and PCD patterns of 77 PCD genes in control and AD cases. Expression analysis revealed that these 77 PCD-related genes exhibited specific patterns, including apoptosis, anoikis, autophagy, and lysosome-dependent cell death (**Figure 2F**), suggesting their close association with AD pathology.

### Development of programmed cell death signature based on an integrated ML-based framework

**Figure 3:**
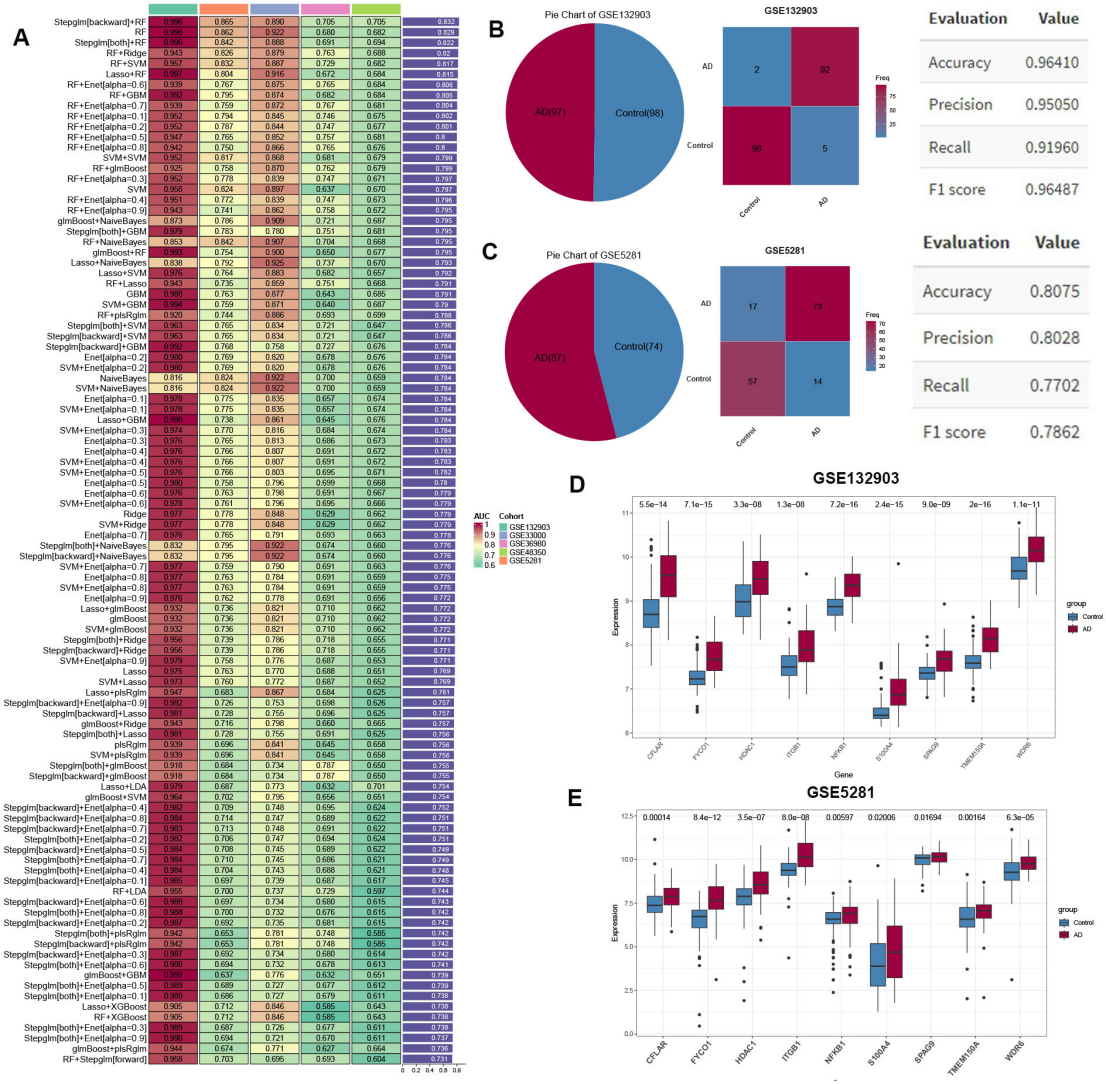
Machine learning-driven PCD signature (PCDS) construction. **(A)** Performance metrics (AUC) of 134 algorithmic models trained on 77 PCD genes across discovery/validation cohorts. **(B, C)** Predictive accuracy matrices for PCDS classification in training **(B)** and independent validation **(C)** datasets (GSE5281). **(D, E)** Violin plots contrasting expression profiles of nine PCDS hub genes between AD and controls in GSE132903 **(D)** and GSE5281 **(E)**.

Through cross-dataset intersection analysis of five independent AD) cohorts (GSE132903, GSE33000, GSE36980, GSE48350, GSE5281), we derived a consensus set of 70 PCD-associated genes for signature development. GSE132903 was designated as the discovery cohort for model training, while GSE33000, GSE36980, GSE48350, and GSE5281 served as validation cohorts. Transcriptomic data from these genes were analyzed using a combinatorial ML framework incorporating 134 distinct model configurations derived from 12 ML algorithms. Cross-cohort validation identified the optimal model through maximum mean area-under-curve (AUC) performance. The Stepglm [backward] feature selection method paired with a Random Forest classifier demonstrated superior discriminative performance (mean AUC = 0.832 across all cohorts), as visualized in **Figure 3A**. Notably, a total of 9 PCDS (CFLAR, FYCO1, HDAC1, ITGB1, NFKB1, S100A4, SPAG9, TMEM150A, and WDR6) achieved AUC values exceeding 0.7 across all cohorts, underscoring the robust generalization ability of the PCDS. Using this model, we generated predicted probabilities and labels, along with confusion matrices for both the training cohort (GSE132903) and the test cohort (GSE5281). Beyond AUC scores, we also evaluated accuracy, precision, recall, and F1 score, which collectively indicated high performance metrics, including precision (>0.8), recall (>0.75), and F1 score (>0.75), reaffirming the reliability and diagnostic efficacy of PCDS in distinguishing between AD and control samples (**Figures 3B, C**). The evaluation results of the model on other datasets were shown in **Supplementary Figure S1**. Furthermore, the expression patterns of PCDS between control and AD cases were investigated in GSE132903 and GSE5281 datasets. As illustrated in **Figures 3D, E**, the upregulated expression levels of all nine PCDS were observed in AD samples when compared to control group, highlighting their potential role in AD pathogenesis and their utility in early prediction and treatment.

### External validation of the predictive capability of PCDS

**Figure 4:**
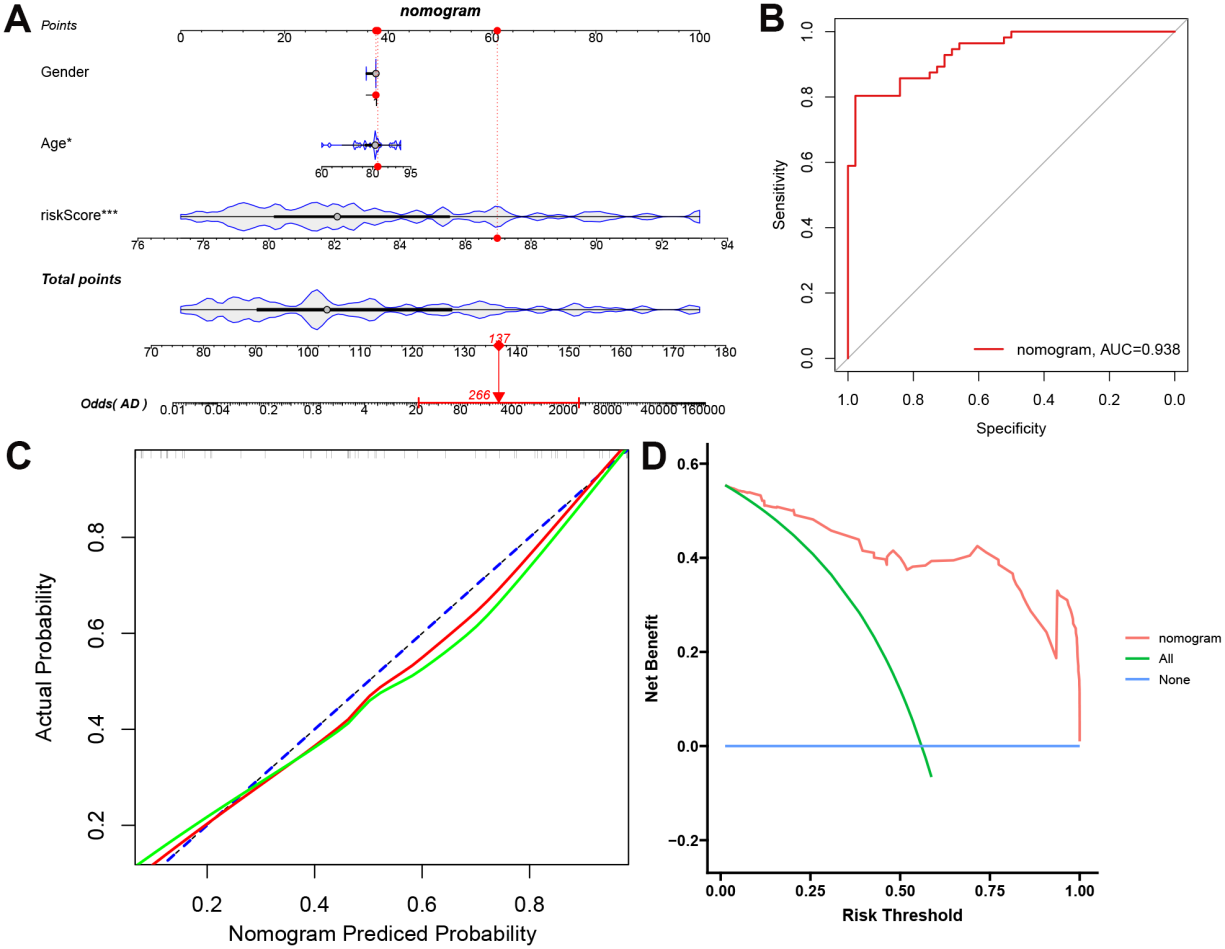
Clinical-translational utility of PCDS. **(A)** Construction of nomogram based on PCDS, gender, and age. **(B)** Receiver operating characteristic (ROC) curve assessing nomogram discrimination capacity. **(C)** The calibration curve of nomogram. **(D)** Decision curve analysis (DCA) quantifying net clinical benefit across risk thresholds to AD patients.

To enhance the clinical generalization ability and optimize the predictive model of PCDS, we constructed a nomogram incorporating clinical features and PCDS using the external validation dataset GSE122063. The uppermost scale of the nomogram represents point assignments for each variable. The subsequent scales correspond to the variables included in the nomogram, including age, sex, and PCDS. By drawing a vertical line from each variable’s value to its corresponding point on the points scale, one can determine the assigned points. For each individual, the total score (fifth scale) is derived by summing these assigned points. Once the total score is calculated, the probability of AD (lowermost scale) can be predicted by locating the total score on the appropriate scale (**Figure 4A**). To evaluate the nomogram’s comprehensive performance, we utilized three commonly employed metrics: discrimination (AUC), calibration, and decision curve analysis (DCA). Notably, our nomogram demonstrated strong diagnostic ability in the GSE122063 cohort (AUC = 0.938) (**Figure 4B**). The calibration curves revealed that the nomogram-predicted probabilities closely aligned with observed probabilities (**Figure 4C**), indicating high accuracy and reliability. Additionally, the DCA curve showed significant clinical net benefit at risk thresholds ranging from 0.4 to 1.0, suggesting its utility in guiding clinical decisions for intermediate- and high-risk AD patients (**Figure 4D**). Overall, the nomogram integrating age, sex, and PCDS performed excellently in predicting AD.

To explore the correlation between PCDS score and key AD-related clinicopathological features, we incorporated an independent dataset, GSE106241, which encompasses multiple clinical phenotypic data from brain tissue samples of AD patients. After removing samples with missing values, a total of 55 AD patients from this dataset was included in our correlation analysis. Our analysis revealed significant positive correlations between the PCDS and several important indicators of AD progression and pathology, including braak stage (cor=0.47, p<0.001), Aβ42 levels (cor=0.28, p=0.033), α-secretase (cor=0.35, p=0.0098), β-secretase (cor=0.59, p<0.001), and γ-secretase (cor=0.45, p<0.001) (**Supplementary Figure S2**), suggesting a higher PCDS may contribute to AD progression.

### Complex relationship between PCDS and biological pathways and immune microenvironment

**Figure 5:**
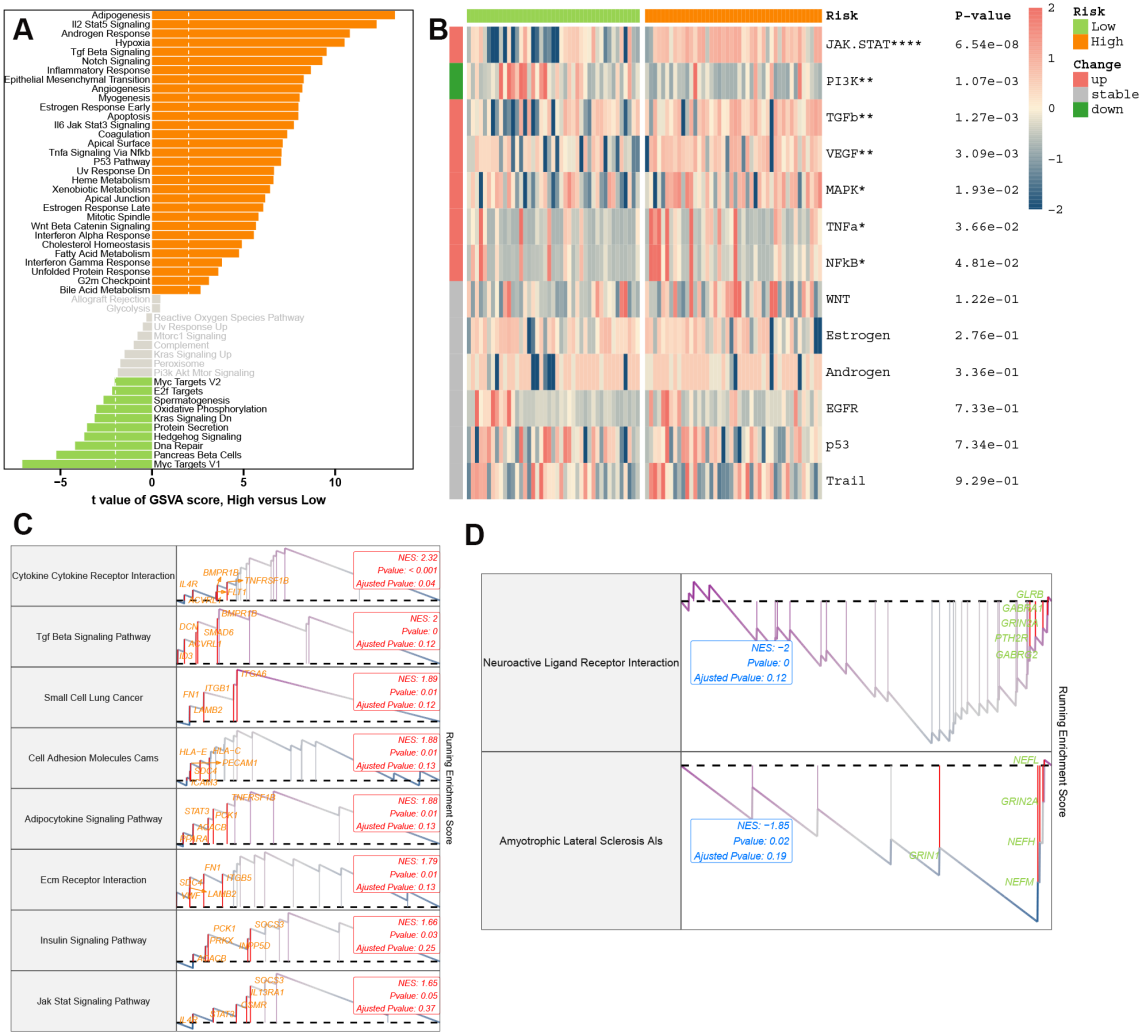
Molecular landscapes stratified by PCDS. **(A)** Gene Set Variation Analysis (GSVA) highlighting pathway activity disparities between PCDS subgroups. **(B)** PROGENy-inferred specific pathway activities between the low- and high-PCDS groups. **(C, D)** Gene Set Enrichment Analysis (GSEA) of upregulated oxidative stress **(C)** and downregulated synaptic plasticity **(D)** pathways in high-PCDS cohorts.

AD patients were stratified into high-PCDS and low-PCDS groups based on the median PCDS score. To elucidate the underlying pathway mechanisms differentiating these groups, we conducted three enrichment analyses. GSVA using hallmark gene sets revealed that high-PCDS patients exhibited upregulation in various pathways related to immunology, inflammation, metabolism, signaling, and proliferation. In contrast, low-PCDS patients showed enrichment in pathways associated with MYC and E2F target genes, oxidative phosphorylation, KRAS signaling, protein secretion, hedgehog signaling, and DNA repair (**Figure 5A**). Moreover, high-PCDS patients exhibited elevated levels of JAK-STAT, MAPK, TGFβ, and NF-κB signaling pathways compared to low-PCDS patients. Conversely, the PI3K signaling pathway was more pronounced in low-PCDS patients (**Figure 5B**). GSEA further demonstrated that high-PCDS patients were significantly associated with cytokine-cytokine receptor interaction, TGF-beta signaling, cell adhesion molecules (CAMs), adipocytokine signaling, ECM-receptor interaction, and insulin signaling pathways (**Figures 5C, D**).

**Figure 6:**
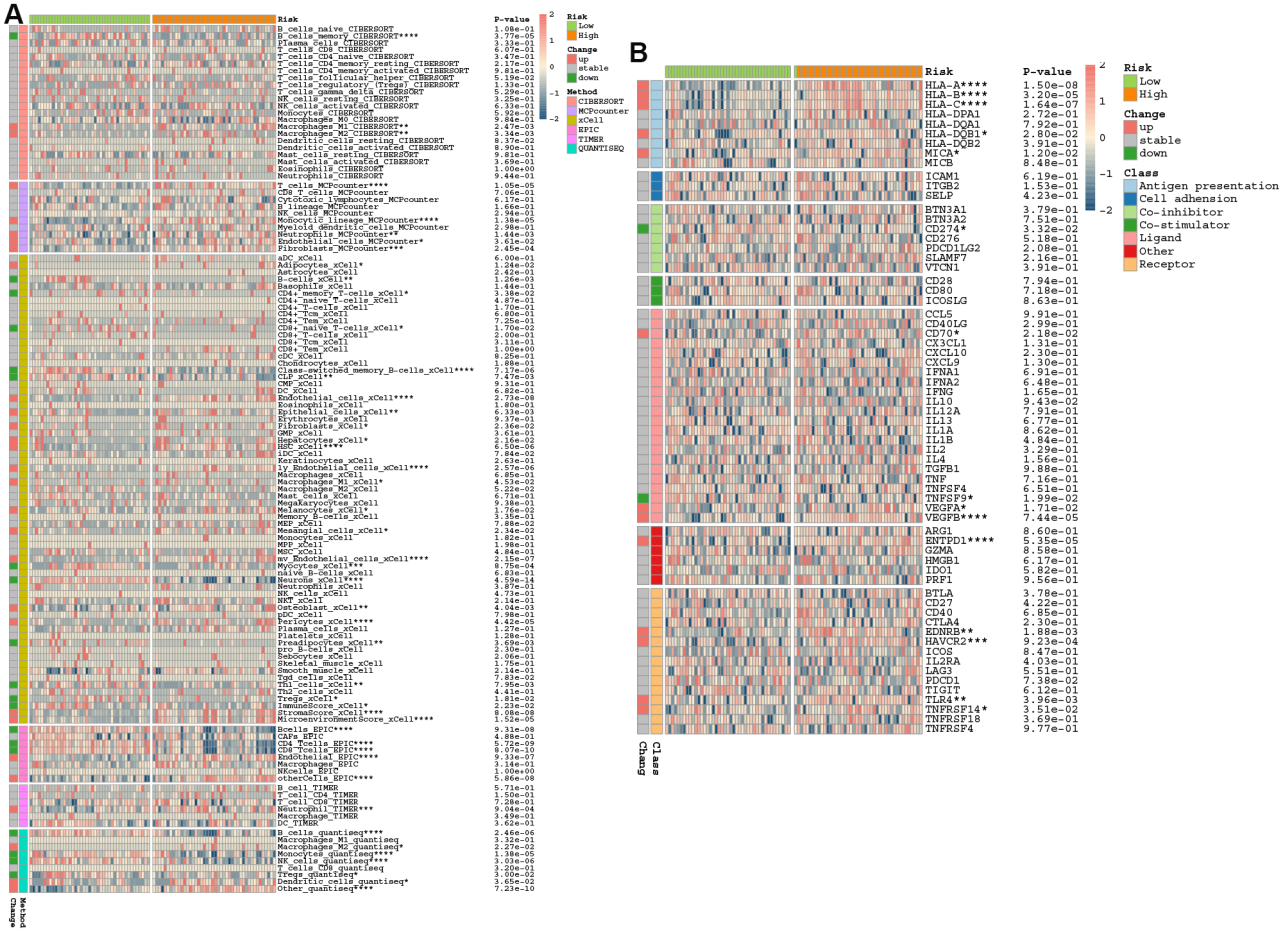
PCDS-associated neuroimmune remodeling. **(A)** Comprehensive analysis of immune infiltration differences (CIBERSORT/MCP-counter/xCell/EPIC/Quantiseq) across PCDS subgroups. **(B)** Differential immune checkpoint between PCDS subgroups.

We subsequently analyzed the immune landscape of both high- and low-PCDS groups using various algorithms (**Figure 6A**). The high-PCDS group exhibited significantly higher infiltration scores for immune cells, including macrophages, T cells, monocytes, neutrophils, pericytes, and dendritic cells. In contrast, the low-PCDS group showed a preference for activating B cells, CD4+ memory T cells, myocytes, preadipocytes, TH1 cells, and regulatory T cells (Tregs). Given the critical role of immune checkpoint molecules in the immune microenvironment, we compared the relative expression levels of immune regulatory genes between distinct PCDS subgroups. High-PCDS patients demonstrated elevated expression of antigen presentation and receptor molecules (**Figure 6B**). These findings suggest that PCDS may influence the pathogenesis of AD by modulating multiple key pathways and immune functions.

### Exploration of PCDS distribution and pseudotime dynamics across microglia transformation

**Figure 7:**
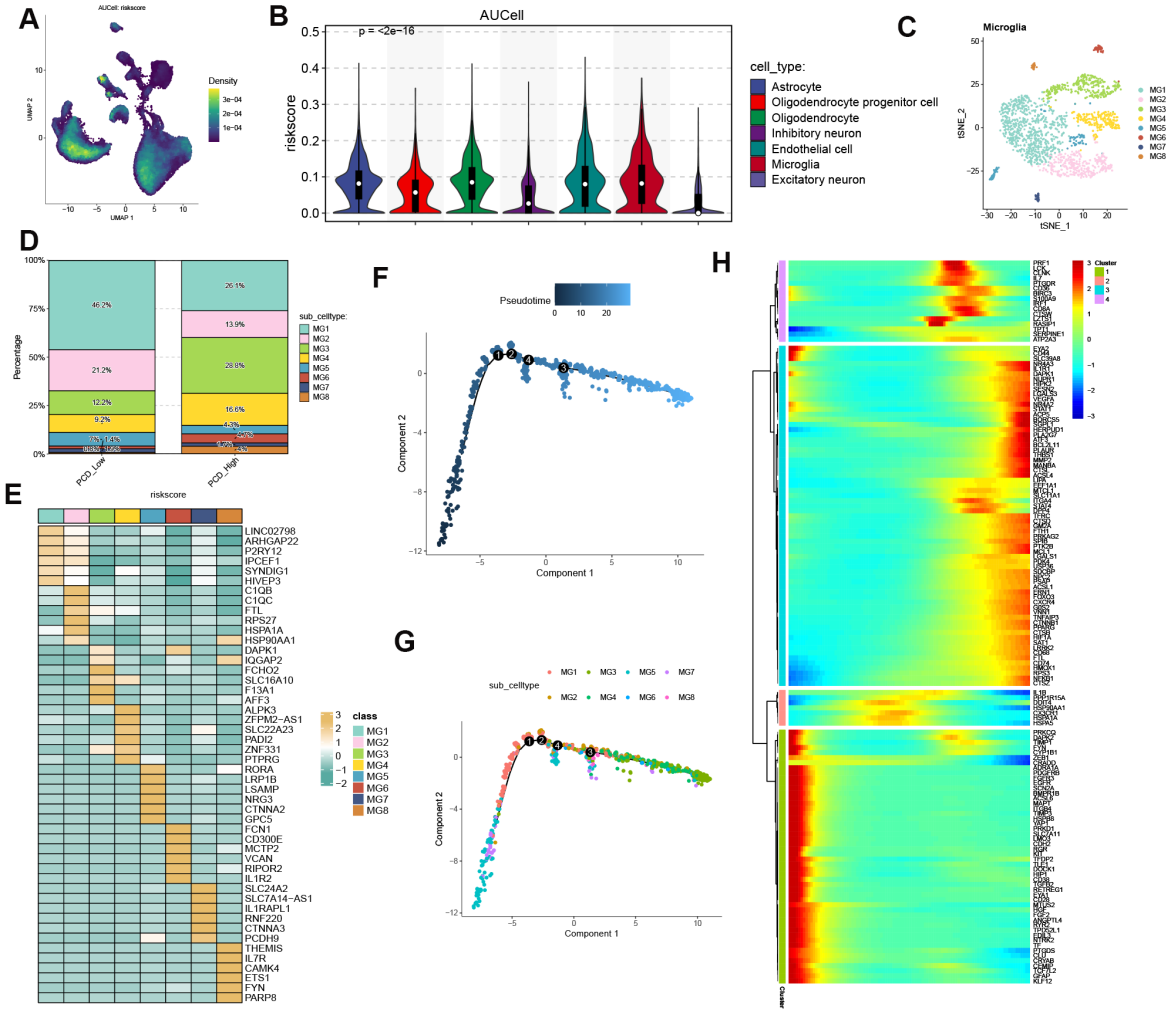
Single-cell resolution of PCDS trajectories. **(A)** UMAP projection of PCDS computed via AUCell scoring. **(B)** Cell-type-specific PCDS enrichment profiles. **(C)** Microglial subpopulation clustering. **(D)** Microglial subtype proportion shifts between PCDS subgroups. **(E)** Top differentially expressed markers across microglial states. **(F–H)** Pseudotemporal ordering of microglial activation trajectories with branch-specific gene dynamics.

To elucidate the role of PCDS in AD pathogenesis, we analyzed the distribution of PCDS across multiple cell types using scRNA transcriptome data from GSE157827. We identified higher PCDS scores in astrocytes, oligodendrocytes, endothelial cells, and microglia (**Figures 7A, B**). Given the critical role of microglia as immune cells in brain tissue, we focused on this cell type for further analysis. Microglia were clustered into eight distinct subtypes (MG1-8) (**Figure 7C**). Based on the 75th percentile of the PCDS score, we categorized microglia from AD patients into high- and low-PCDS groups. In the low-PCDS group, MG1 was the predominant subtype, comprising 46.2% of cells, followed by MG2 (21.2%), MG3 (12.2%), MG4 (9.2%), MG5 (7%), MG6 (1.4%), MG7 (0.8%), and MG8 (1.9%). Conversely, in the high-PCDS group, the proportions of MG3 (28.8%) and MG4 (28.8%) significantly increased, while MG1 (26.1%) and MG2 (13.9%) decreased substantially. Notably, MG6 showed a modest increase (4.7%) and MG5 decreased to 4.3% (**Figure 7D**). The top six differentially expressed genes across all microglia subtypes are illustrated in **Figure 7E**.

Additionally, we investigated the dynamics of PCDS expression patterns using Monocle analysis for trajectory inference. As anticipated, MG3 and MG4 predominantly appeared at the end of differentiation with higher pseudotime values, whereas microglia from MG5, MG1, and MG2 were mainly distributed at the beginning of differentiation (**Figures 7F, G**). Next, we explored whether PCDS undergoes dynamic changes during microglial transformation by clustering PCD-related genes that were differentially expressed along the trajectory. We identified four distinct kinetic patterns. Notably, some genes, including PRKCQ, DAPK2, TIMP1, FYN, CYP1B1, and ZEB1, exhibited a similar trend during the initial phase. In contrast, genes such as PRF1, LCK, NR4A3, IL1R1, ACSL4, CTSD, FTH1, and FOXO3 showed activity in intermediate and late stages (**Figure 7H**). Collectively, these results suggest that PCDS may drive the progression of AD.

### Identification of crucial cellular communication affected by PCDS

**Figure 8:**
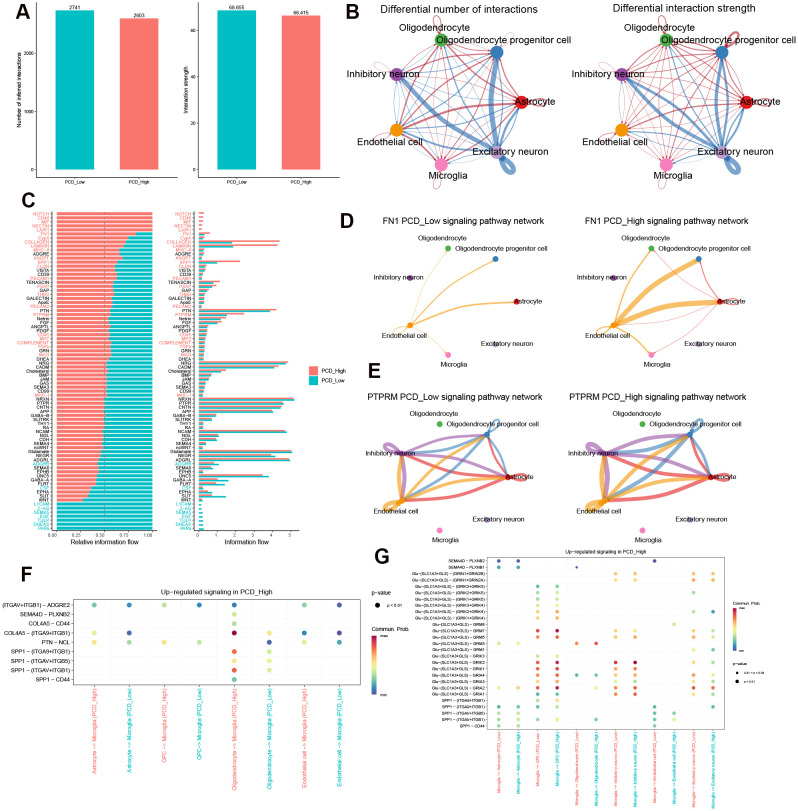
Cell-cell communication networks modulated by PCDS. **(A)** Quantitative comparison of intercellular interaction frequency/strength. **(B)** Ligand-receptor network topology alterations between subgroups. **(C)** Pathway-centric information flux disparities. Red represents pathways enriched in high-PCDS group, while green represents pathways enriched in low-PCDS group. **(D, E)** The inferred FN1 **(D)** and PTPRM **(E)** signaling networks. **(F, G)** Dotplots of significant cytokine ligand (source) -receptor (target) interactions between microglia and other cells discovered using CellChat. Color represents communication probabilities, and the bubble size represents p-value of the ligand-receptor pairs between microglia and other cells.

We conducted a comprehensive analysis of cell interactions across distinct cell types in low-PCDS and high-PCDS groups using the CellChat method. The high-PCDS group exhibited reduced interaction numbers and strengths compared to the low-PCDS group (**Figure 8A**). Specifically, the signals from excitatory neuron to inhibitory neuron, oligodendrocyte progenitor cell, and astrocyte were diminished, whereas those between astrocyte, microglia, endothelial cell, and oligodendrocyte were significantly increased in high-PCDS patients (**Figure 8B**). Based on the whole signaling patterns and relative information flow, we found that high-PCDS was likely associated with upregulation of several signaling pathways, including NOTCH, CD46, MIF, NECTIN, LAIR1, FN1, CypA, COLLAGEN, LAMININ, MHC-II, ANGPT, GPP1, CLDN, PECAM1, PSAP, THBS, PECAM2, PEPRM, CD45, MPZ, COMPLEMENT, TGFβ, and MAG. Conversely, it inhibited ADGRB, CSF, L1CAM, 2-AG, SEMA5, EGF, TULP, DHEAS, and PARs signaling pathways (**Figure 8C**). Additionally, in the high-PCDS group, FN1 signaling networks from endothelial cell to microglia, astrocyte, oligodendrocyte, and oligodendrocyte progenitor cell, as well as PTPRM signaling networks involving inhibitory neuron, endothelial cell, astrocyte, and oligodendrocyte progenitor cell, were more pronounced. For instance, endothelial cell-secreted FN1 signaling was significantly sensed by astrocyte and oligodendrocyte progenitor cell in the high-PCDS group but less in the low-PCDS group (**Figures 8D, E**). Detailed analysis of cell–cell interactions revealed that SPP1, COL4A5, PTN, SEMA4D, and Glu-dependent ligand-receptor pairs were activated by microglia in the high-PCDS group (**Figures 8F, G**).

### S100A4 knockdown improves microglia viability and exerts neuroprotection after AbetaO injury

**Figure 9:**
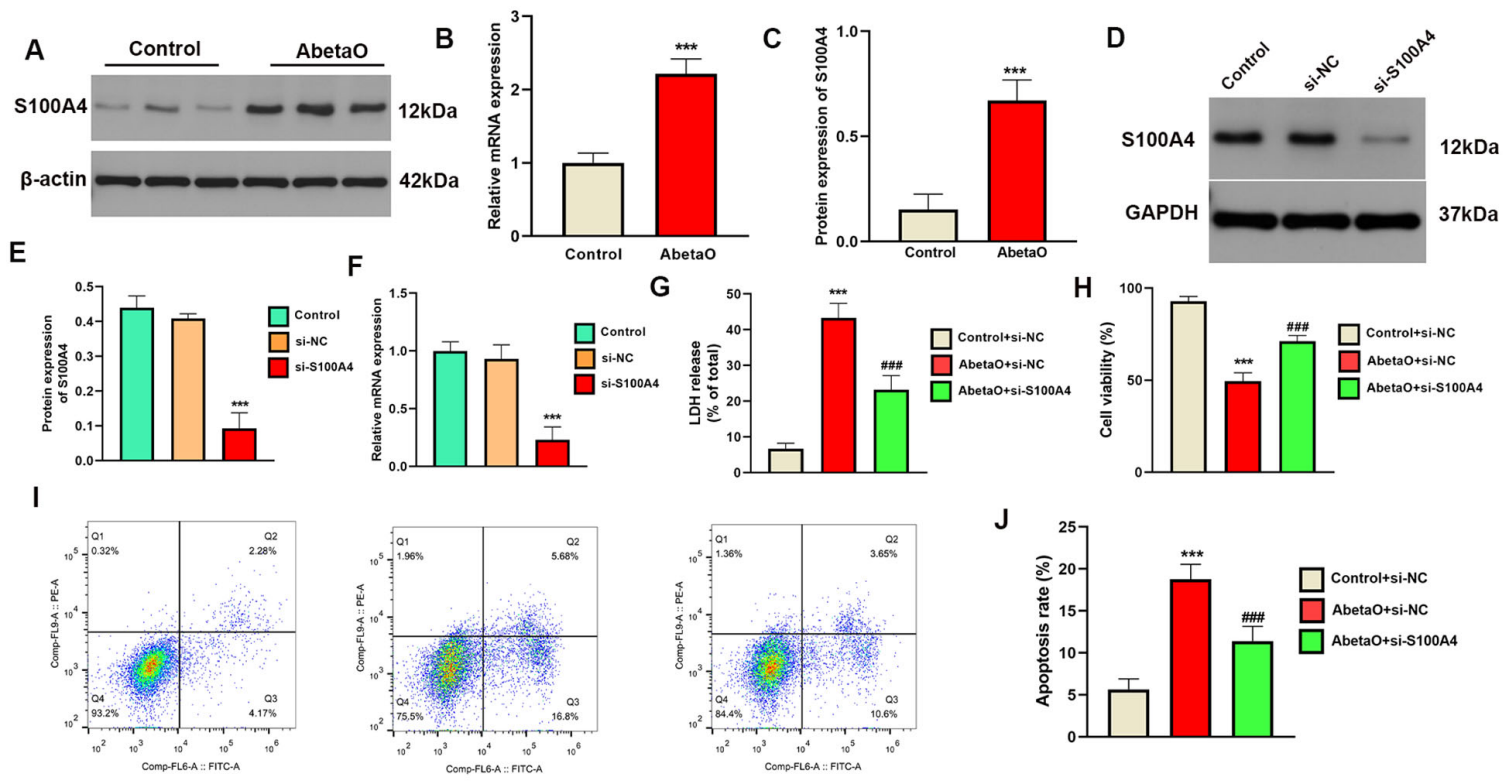
S100A4 knockdown improves microglia viability and exerts neuroprotection after AbetaO injury. **(A-C)** Expression profiles of S100A4 were evaluated by western blot **(A, B)** and qRT-PCR **(C)** (n=3). **(D-F)** The knockdown efficiency of S100A4 was evaluated by western blot **(D, E)** and qRT-PCR **(F)** (n=3). **(G)** CCK-8 viability assay of AbetaO-exposed microglia (n=3). **(H)** LDH cytotoxicity quantification after AbetaO damage (n=3). **(I-J)** Annexin V/PI flow cytometry quantifying apoptosis rates (n=3). Data are presented as mean ± SD. ^***^p<0.001 vs. Control+si-NC; ^###^p< 0.001 vs. AbetaO+ si-NC.

To translate our PCDS findings into functional insights, particularly concerning microglial contributions to AD, we firstly assessed the expression patterns of all nine PCDS genes across different cell types at single-cell level. We found S100A4 displayed a more restricted and prominent expression pattern, which was predominantly observed in microglia and, to some extent, in endothelial cells. In contrast, the other eight PCDS genes generally showed more widespread expression across various cell types, including neurons, astrocytes, oligodendrocytes, and endothelial cells. In addition, our prior bulk RNA-seq analyses have demonstrated that S100A4 was highly expressed in AD brain samples compared with normal brain tissues. Furthermore, limited literature is available to clarify the pathological role of S100A4 in AD. Therefore, we focused on the S100A4, a critical model gene in this context. *In vitro* experiments revealed that both S100A4 protein and mRNA levels were significantly elevated in AbetaO-induced BV2 microglia (**Figures 9A–C**). To investigate the functional role of S100A4 in AD, we knocked down its expression in BV2 cells using specific siRNA. The western-blot and qRT-PCR experiments were performed to confirm the efficacy of S100A4 silencing, and the significant reduction in the protein and mRNA expression levels of S100A4 were observed (**Figures 9D–F** **).** Our findings indicate that inhibiting S100A4 significantly increased cell viability, as measured by CCK-8 assays, and reduced LDH release in AbetaO-treated BV2 cells (**Figures 9G, H**). Additionally, flow cytometry analysis showed that S100A4 knockdown partially alleviated AbetaO-induced apoptosis in BV2 cells (**Figures 9I, J**). We next investigated the neuroprotective effects of S100A4 *in vitro*. BV2 cells were co-cultured with AbetaO for 24 hours, and the conditioned medium was then collected to stimulate normal HT-22 hippocampal neuronal cells. As shown in **Supplementary Figure S4**, the percentage of TUNEL+ cells significantly increased in HT-22 neuronal cells treated with BV2 conditioned medium containing AbetaO (AbetaO-CM), but was significantly reduced after S100A4 knockdown treatment (AbetaO+si-S100A4-CM) (**Supplementary Figure S3**). These results suggest that S100A4 plays a crucial role in improving microglia viability and prevented neuron from apoptosis in the context of AD.

### S100A4 knockdown suppresses AbetaO-induced pro-inflammatory microglial activation

**Figure 10:**
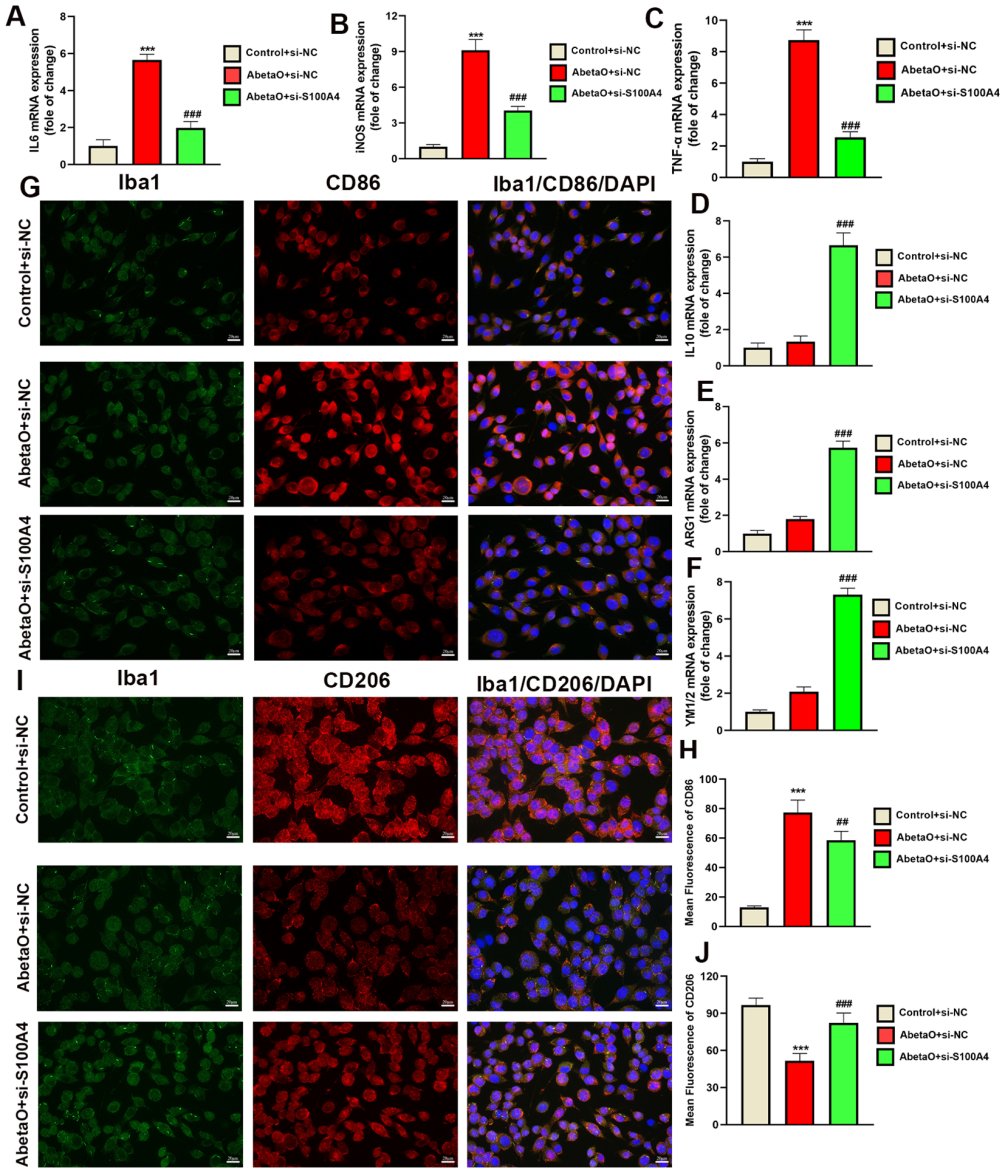
S100A4 knockdown modulates AbetaO-induced microglial activation. **(A–F)** Quantitative RT-PCR analysis of IL6 **(A)**, iNOS **(B)**,TNFα **(C)**, IL10 **(D)**, ARG1 **(E)**, and YM1/2 **(F)** (n=4). **(G–J)** Immunofluorescence staining and quantitative results of CD86 (pro-inflammatory marker, red) and CD206 (anti-inflammatory marker, red) in BV2 microglia (N=4). Data are presented as mean ± SD. ^***^p<0.001 vs. Control+si-NC; ^##^p< 0.01, ^###^p< 0.001 vs. AbetaO+ si-NC.

To investigate the effects of S100A4 on the phenotype and function of microglia following AbetaO treatment, we examined inflammatory cytokine secretion and microglial phenotypes *in vitro*. RT-qPCR results demonstrated that S100A4 knockdown markedly reduced the expression of genes typically associated with pro-inflammatory responses (IL-6, iNOS, and TNF-α) and enhanced the expression of anti-inflammatory genes (IL-10, ARG1, and YM1/2) in AbetaO-induced BV2 cells (**Figures 10A–F**). Additionally, immunofluorescence analysis revealed that AbetaO stimulation significantly increased the fluorescence intensity of the cell surface marker CD86, which is often upregulated during pro-inflammatory microglial activation. Conversely, a reduced fluorescence intensity was observed for CD206, a marker frequently associated with alternative activation states and phagocytosis. Knockdown of S100A4 attenuated CD86 expression and elevated CD206 expression in BV2 cells exposed to AbetaO (**Figures 10G–J**). These findings suggest that inhibiting S100A4 promotes a shift in microglial activation from a predominantly pro-inflammatory state towards one characterized by increased expression of anti-inflammatory markers in AbetaO-exposed BV2 cells.

### S100A4 knockdown inhibits AbetaO-induced oxidative stress

**Figure 11:**
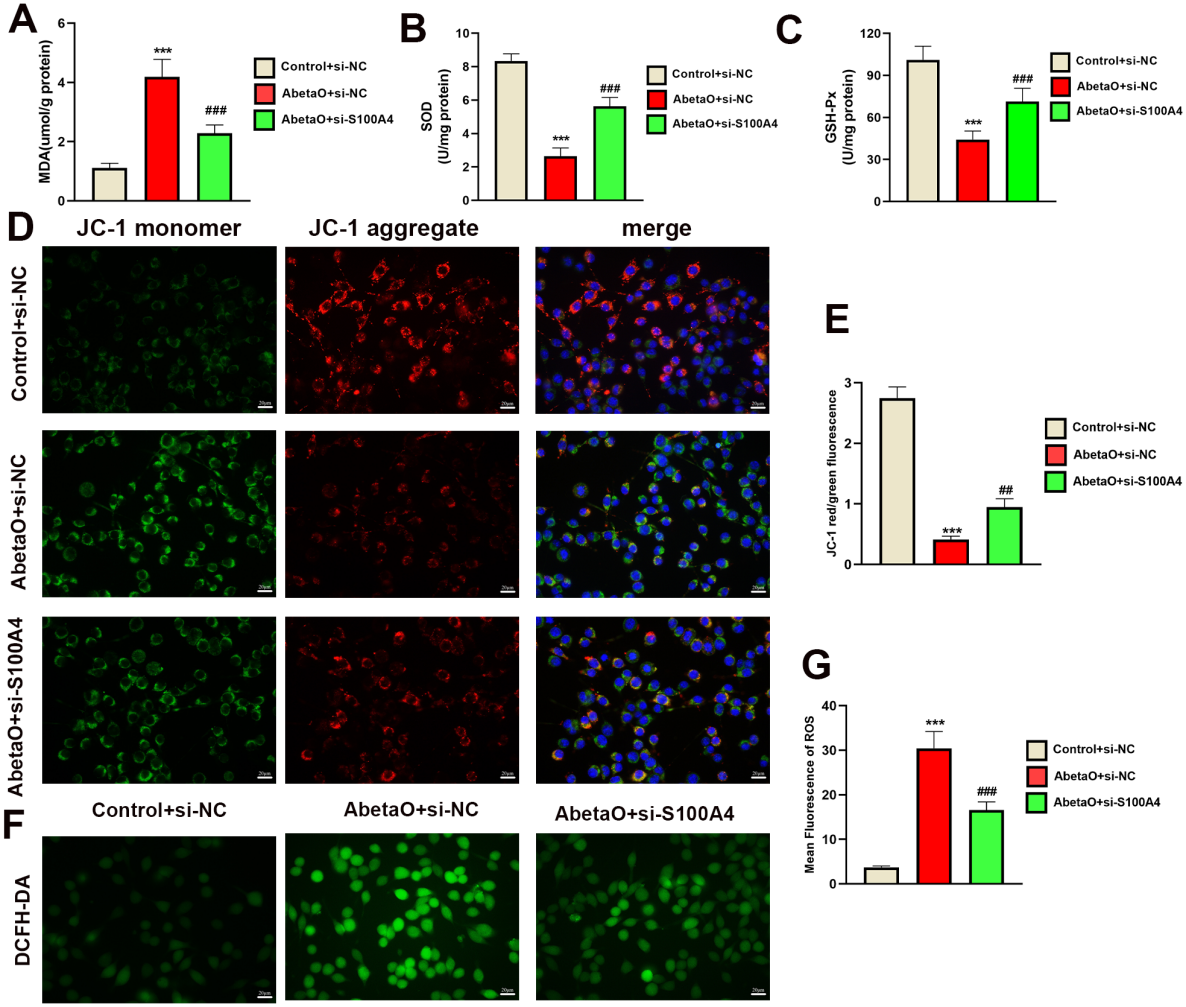
S100A4 knockdown inhibits AbetaO-triggered redox imbalance. **(A–C)** Inhibition of S100A4 significantly modulated redox homeostasis in AbetaO-exposed BV2 microglia, evidenced by reduced MDA concentrations **(A)**, enhanced SOD activity **(B)**, and elevated GSH-Px enzymatic function **(C)** (n = 3) **(D, E)** The effects of S100A4 knockdown on the MMP of BV2 microglia with AbetaO stimulation were assessed using the Immunofluorescence microscopy. Fluorescence imaging revealed JC-1 aggregates (red, polarized mitochondria) and monomers (green, depolarized mitochondria) (scale bar: 20 μm), with quantitative analysis reflecting the red/green intensity ratio (n = 3). Data are presented as mean ± SD. ^***^p<0.001 vs. Control+si-NC; ^##^p< 0.01, ^###^p< 0.001 vs. AbetaO+ si-NC.

To investigate the impact of S100A4 on AbetaO-induced oxidative stress and mitochondrial integrity, we measured key oxidative markers and mitochondrial membrane potential (MMP). As shown in **Figures 11A–C**, AbetaO stimulation led to a significant imbalance in redox homeostasis, evidenced by reduced activities of the antioxidant enzymes SOD and GSH-Px, alongside a marked increase in the lipid peroxidation product MDA. Concurrently, AbetaO exposure triggered a substantial elevation in intracellular reactive oxygen species (ROS) levels (**Figures 11F, G**). This heightened oxidative stress state is known to directly compromise mitochondrial health. Indeed, we observed a significant decrease in MMP (indicated by a reduced red/green JC-1 fluorescence ratio) in AbetaO-treated cells, reflecting mitochondrial dysfunction (**Figures 11D, E**). Importantly, knockdown of S100A4 effectively counteracted these detrimental effects. Inhibition of S100A4 not only suppressed the AbetaO-induced surge in ROS levels but also partially restored SOD and GSH-Px activities and reduced MDA accumulation (**Figures 11A–C, F–G**). Crucially, this alleviation of oxidative stress by S100A4 knockdown was associated with a significant restoration of MMP (**Figures 11D, E**). These results strongly suggest that S100A4 contributes to AbetaO-induced mitochondrial dysfunction by exacerbating oxidative stress, and its inhibition protects mitochondria, at least in part, by mitigating this oxidative damage.

## Discussion

Despite advancements in diagnosis and treatment, the improvement in Alzheimer’s disease (AD) prognosis remains limited, therefore bringing significant medical burdens globally. Many patients receive diagnoses at advanced stages, which can hinder treatment efficacy. Consequently, early diagnosis and intervention are crucial for enhancing outcomes. However, current biomarkers—such as the Aβ42/Aβ40 ratio, pTau181, pTau217, and NFL levels in blood (38, 39) are still far from the standard of ideal biomarkers diagnosed by AD diagnosis and deserve to be further explored.

Given the crucial role of PCD in in various disease progressions (40–42), we utilized a scRNA-seq dataset (comprising nine controls and twelve AD cases) to investigate pan-cell death patterns. Results indicated higher levels of PCD in microglia cells, with eleven distinct PCD patterns, excluding pyroptosis, parthanatos, alkaliptosis, oxeiptosis, and paraptosis, significantly activated in AD-affected microglia. Recent studies have reported that multiple signal transduction pathways, including p53, KRAS, NOTCH signaling, hypoxia, and metabolic reprogramming, regulate the initiation of certain types of PCD (43). The potential effects and mechanisms of these distinct PCD forms in AD have been elucidated. For instance, ferroptosis has been implicated in abnormal microglia activation, a hallmark of chronic neuroinflammation in AD (44). Abnormal activation of microglia leads to the release of pro-inflammatory cytokines and reactive oxygen species (ROS), contributing to neurodegeneration (45). The metal ion chelator deferoxamine (DFO) is shown to inhibit the accumulation of iron in the brain tissues of AD animals, followed by the reduction of Aβ plaque formation (46), suggesting that targeting ferroptosis-related pathways, such as iron metabolism, lipid metabolism, and the GPX4 antioxidant system, could be a promising therapeutic breakthrough for AD (19). Necroptosis has been identified as a key player in AD pathogenesis. Aβ aggregates promote neuronal necroptosis via the RIPK1-MLKL axis, forming a cascade effect (47). Additionally, tau activates the RIPK1/RIPK3/MLKL and NF-κB signaling pathways, mediating necrotic apoptosis and inflammation, which drives cell death (48). Pharmacological and genetic inhibition of RIPK1/3 and MLKL have effectively ameliorated pathological changes and cognitive deficits in AD animal models (49). Cuproptosis, a novel form of PCD, has been shown to promote AD development. Bioinformatics analysis has identified cuproptosis-related genes and developed predictive models for AD (50). In addition, cuproptosis can promote the activation of the NF-κB signaling pathway and the release of inflammatory factors, thereby driving AD progression (51). Increased copper exporters (APP7A/B), GSH, and antioxidants may serve as anti-cuproptosis strategies targeting AD (52). However, limited information is available on the roles of anoikis, NETotic cell death, entotic cell death, and disulfidoptosis in AD. Using WGCNA and upregulated differentially expressed genes (DEGs), we identified 77 PCD-related genes significantly upregulated in AD, suggesting their crucial role in driving AD.

Machine learning techniques have been widely employed for the early diagnosis of various cardiovascular and cerebrovascular diseases (24–26). These methods have demonstrated promising results in improving diagnostic accuracy and efficiency. However, effectively implementing these models while maintaining high performance remains challenging. Additionally, selecting the most appropriate ML algorithm is a critical decision influenced by various factors such as the problem’s characteristics and researcher preferences. In this study, we curated expression profiles from hundreds of controls and AD case brain tissues across five multicenter cohorts worldwide. and developed a PCDS using an integrative machine learning framework that combines multiple model predictions to achieve higher accuracy. A total of 134 ML integrations were benchmarked and optimized via 10-fold cross-validation, leading to the selection of more effective features and the generation of robust prediction models. Ultimately, the best-performing model, termed PCDS, was derived from the expression of nine genes (CFLAR, FYCO1, HDAC1, ITGB1, NFKB1, S100A4, SPAG9, TMEM150A, and WDR6), combining Stepglm [backward] and RF. This model exhibited the highest average Area Under the Curve (AUC) score of 0.832 across five independent cohorts. We further developed a nomogram based on the PCDS model, demonstrating its strong predictive ability and clinical utility through rigorous evaluation criteria, including AUC, calibration curves, and DCA. Our findings suggest that PCDS can serve as a valuable tool for guiding therapeutic decisions and early diagnosis.

Given the substantial treatment challenges associated with AD, we aim to explore the pathological role and potential mechanisms of PCDS in AD, which may provide novel insights for therapeutic strategies. We stratified AD patients into two distinct risk subgroups to examine the molecular signatures associated with different risk levels. Pathway enrichment analyses revealed that high PCDS correlates with disease progression, characterized by significant enrichment of hypoxia, apoptosis, inflammatory response, p53 signaling, TNFα signaling, interferon response, and other pathways previously implicated in AD pathogenesis (53–56). Conversely, low PCDS patients exhibited involvement in protective pathways such as oxidative phosphorylation, protein secretion, and DNA repair. Consistent with these findings, Gene Set Enrichment Analysis (GSEA) demonstrated increased activity of the JAK/STAT, TGFβ, TNFα, and NFκB pathways in the high PCDS group compared to the low PCDS samples. Single-cell RNA sequencing (scRNA-seq) analysis highlighted elevated PCDS activity in microglia. Subsequent analysis identified eight critical microglial subpopulations, with MG1 and MG2, associated with homeostatic and stress functions, significantly augmented in the low-PCDS group. Meanwhile, MG3, MG4, and MG6 subtypes, linked to phagocytic, lipid-processing, and pro-inflammatory functions, were more prevalent in high-PCDS patients. These characteristics show a striking resemblance to the well-documented “Disease-Associated Microglia” (DAM) phenotype, a specific microglial state observed in proximity to amyloid plaques in Alzheimer’s disease and other neurodegenerative conditions (57). The DAM program involves the downregulation of homeostatic genes and the upregulation of a specific transcriptional signature, including genes involved in lipid metabolism and phagocytosis, which aligns closely with the profile of our identified MG3/MG4 subtypes. Recent studies suggest that lipid accumulation and peroxidation can activate inflammatory signaling pathways in microglia, promoting the release of inflammatory factors such as IL-1β and TNF-α, thereby contributing to Aβ plaque accumulation (58–60). In the early stages of AD, microglia effectively remove Aβ plaques via the complement system and the release of C1Q and other factors. However, their phagocytic ability diminishes as the disease progresses (61, 62). Furthermore, the transition from homeostatic microglia to this DAM state is critically dependent on the TREM2 (Triggering Receptor Expressed on Myeloid cells 2) signaling pathway (63, 64). TREM2 is a key genetic risk factor for late-onset AD, and it functions as a sensor for pathological changes, including binding to lipids and amyloid-beta, thereby triggering the protective activation of microglia (65, 66). Therefore, our finding that a high PCDS score correlates with an expansion of these DAM-like subtypes (MG3/MG4) suggests that our signature may capture the activation of this crucial TREM2-DAM axis. This contextualizes the PCDS within a core pathogenic mechanism of AD and reinforces the link between programmed cell death pathways and the neuroinflammatory response mediated by microglia.

While previous studies have successfully developed prognostic and diagnostic models for Alzheimer’s disease by focusing on individual PCD pathways—such as cuproptosis (50), ferroptosis (67), pyroptosis (68), autophagy (69), and PANoptosis (70)—our study introduces several significant advancements. Firstly, our PCDS was not limited to a single or several PCD modality but was derived from a comprehensive set of 17 different PCD patterns, providing a more holistic view of the cell death landscape in AD. Secondly, the robustness of our PCDS is underscored by its rigorous validation across five independent cohorts, a more extensive validation than is often reported in initial signature-development studies. A key methodological innovation of our work is the application of an integrated machine learning framework that benchmarked 134 model permutations to identify the optimal algorithm, rather than relying on a single pre-selected method. This data-driven approach enhances confidence in the predictive power and stability of the resulting 9-gene signature. Crucially, we have bridged the gap between a transcriptomic signature and its cellular basis by linking the PCDS score directly to microglial subtype shifts at the single-cell level, a layer of mechanistic detail not present in previous models. Finally, by proceeding to the functional validation of a key signature gene, S100A4, we provide tangible experimental evidence for the signature’s biological relevance, demonstrating its role in microglial viability, neuroinflammation, and oxidative stress. This multi-layered approach: from a broad, multi-cohort signature development to single-cell analysis and specific gene validation, collectively enhances the robustness and mechanistic insight of our model compared to prior work in the field.

Of the nine genes involved in developing PCDS, S100A4, a member of the S100 protein family, has been implicated in regulating various cellular processes, including proliferation, migration, apoptosis, calcium homeostasis, metabolism, and inflammation (71). S100A4 has been shown to be significantly upregulated in idiopathic pulmonary fibrosis (72). In multiple sclerosis, S100A4 overexpression promotes microglial polarization of pro-inflammatory type via the TLR4/NF-κB pathway, initiating neuroinflammation (73). Notably, increased S100A4 expression is observed in kidney fibrosis, and its pharmacological inhibition markedly reduces fibroblast activation. Conversely, another study found that S100A4 overexpression exerts anti-apoptotic effects by upregulating the AKT signaling pathway in mice with retinal ischemia-reperfusion injury (74), highlighting the complex roles of S100A4 in different diseases. Therefore, we aim to investigate the specific effects of S100A4 in Alzheimer’s disease.

Oxidative stress and consistent neuroinflammation are established as critical pathological factors in the development of AD (53, 75–77). Emerging studies indicate that microglia, the resident macrophages of the central nervous system, play a pivotal role in maintaining brain homeostasis by regulating oxidative stress and neuroinflammation (78–80). Microglial activation is a complex response to stress or injury. Activated microglia can release pro-inflammatory cytokines and peroxides, potentially promoting neurodegeneration, or they can exhibit functions associated with anti-inflammatory properties and immune homeostasis (81, 82). Therefore, we mainly focus on the effect of S100A4 on the function of microglia following AD. In our study, we investigated the impact of S100A4 on microglial function in AD, demonstrating that S100A4 knockdown facilitated a shift in the microglial activation profile, reducing the expression of pro-inflammatory mediators and enhancing markers associated with anti-inflammatory in BV2 cells following AbetaO treatment. While our results demonstrate a clear shift away from the AbetaO-induced pro-inflammatory phenotype upon S100A4 knockdown, we acknowledge that the inclusion of classical M1/M2 polarizing agents as positive controls would further strengthen these findings. In addition, our *in vitro* experiments further elucidated the mechanisms by which S100A4 influences microglial responses to AbetaO. We found that S100A4 knockdown significantly attenuated AbetaO-induced oxidative stress, as evidenced by reduced ROS production and lipid peroxidation (MDA), alongside enhanced antioxidant enzyme (SOD, GSH-Px) activities. It is well established that excessive oxidative stress is a primary driver of mitochondrial damage (83–85). Mitochondria are both major producers of ROS and primary targets of ROS-mediated injury, creating a vicious cycle that can lead to the collapse of the mitochondrial membrane potential (MMP) -a critical indicator of mitochondrial integrity and function. In line with this, our study demonstrated that the AbetaO-induced oxidative stress was directly linked to a significant loss of MMP in microglia. Notably, the protective effect of S100A4 knockdown against oxidative stress translated directly into a significant preservation of MMP. This finding emphasizes a key mechanistic link: S100A4 exacerbates AbetaO-induced microglial injury by promoting an oxidative environment that impairs mitochondrial function. Conversely, inhibiting S100A4 confers protection by restoring redox balance, thereby maintaining mitochondrial integrity and MMP, which is crucial for cell survival and function. This mitochondrial protection likely contributes to the observed improvements in cell viability and reduced apoptosis in S100A4-deficient microglia exposed to AbetaO.

Although the potential impact of PCDS on predicting and treating AD has been confirmed, several limitations must be acknowledged. First, the retrospective nature of the sample set and its relatively small size necessitate further validation through prospective, large-scale multicenter studies to ensure the generalizability of PCDS. Second, the relatively small sample size of the GSE106241 cohort is a limitation for the correlation analyses. The correlation between PCDS score and clinicopathological features should be interpreted with caution and warrant validation in larger, independent cohorts. Third, Future studies would be strengthened by the inclusion of these standard positive controls (LPS/IL-4) to better contextualize the magnitude of the phenotypic shift we observed. Finally, the specific biological mechanisms by which S100A4 influences microglial function require further exploration through additional *in vivo* and *in vitro* experiments.

## Conclusion

Our study identified a novel PCDS comprising nine genes (CFLAR, FYCO1, HDAC1, ITGB1, NFKB1, S100A4, SPAG9, TMEM150A, WDR6) that robustly predicts and classifies Alzheimer’s disease (AD). The PCDS demonstrated high accuracy across multiple cohorts, with an average AUC of 0.832. Functional analyses revealed that S100A4 knockdown improved microglial viability, reduced apoptosis, suppressed M1 polarization, and mitigated oxidative stress induced by amyloid-beta oligomers. These findings highlight the potential of PCDS as a biomarker for early AD diagnosis and suggest S100A4 as a therapeutic target.

## Data Availability

The datasets generated for this study can be found in the https://www.ncbi.nlm.nih.gov/geo/. The names of the repository/repositories and accession number(s) can be found in the article/**Supplementary Material**.

## Supplementary material

Supplementary Figure 1 Model evaluation on other datasets. **(A–C)** The prediction accuracy matrix of PCDS classification and the expression profiles of nine PCDS hub genes in GSE33000, GSE36980, and GSE48350.

Supplementary Figure 2 The PCDS score predicts the progression of AD. **(A–E)** The correlation between PCDS score and key AD-related clinicopathological features, including braak stage, Aβ42 levels, α-secretase, β-secretase, and γ-secretase levels.

Supplementary Figure 3 Knockdown of S100A4 inhibits neuronal apoptosis. **(G–J)** TUNEL staining and quantitative results of the cell apoptosis in HT-22 neuronal cells (N=4). Data are presented as mean ± SD. ^***^p<0.001 vs. Ctrl-CM; ^###^p< 0.001 vs. AbetaO-CM.
