# Supplementary figures and images for: Programmed cell death signatures-driven microglial transformation in Alzheimer’s disease: single-cell transcriptomics and functional validation
Source: Front Immunol. 2025 Jul 25;16:1610717. doi: 10.3389/fimmu.2025.1610717 (PMC12331497; doi:10.3389/fimmu.2025.1610717)

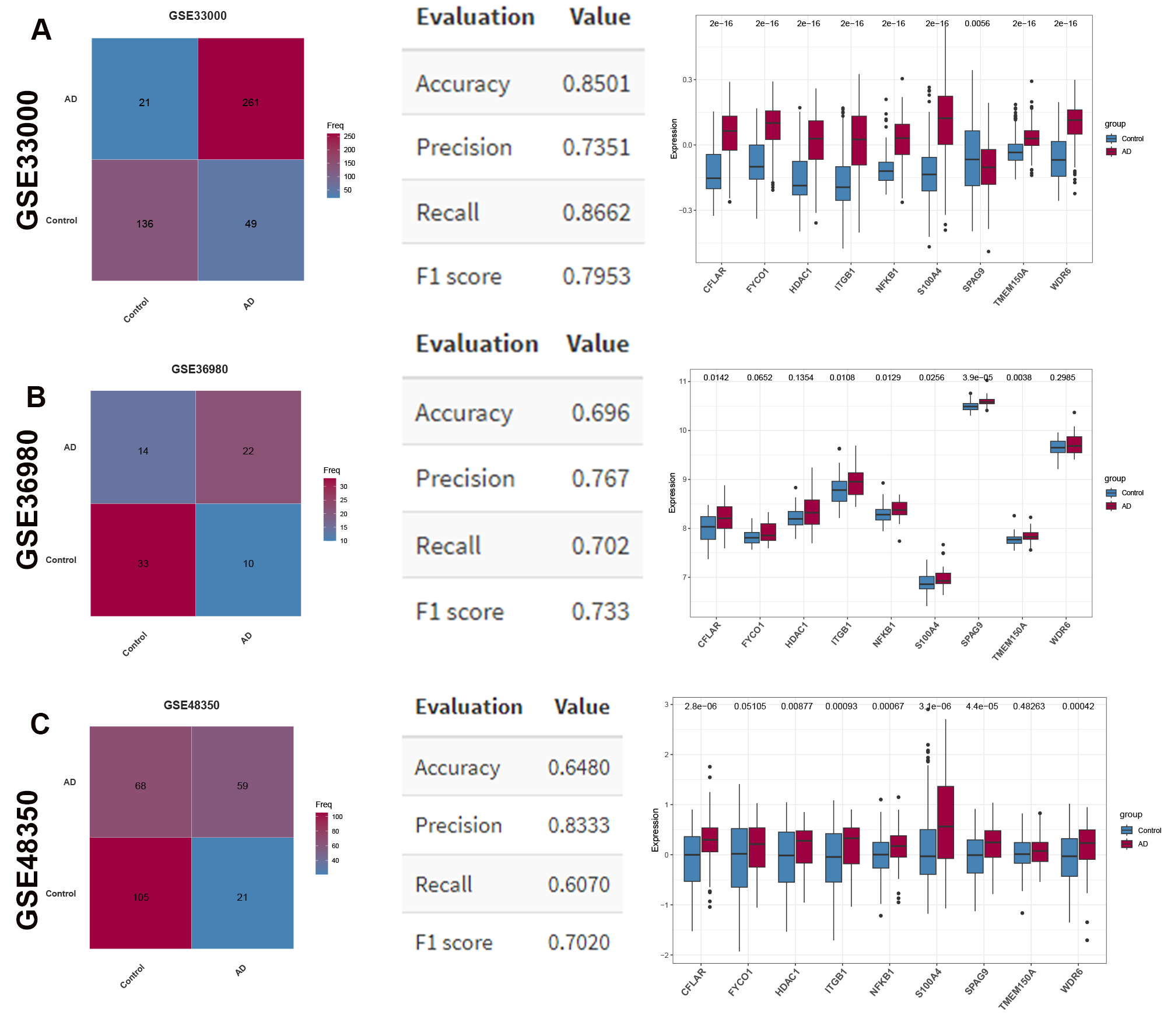

Supplement: Supplementary Figure 1 — Model evaluation on other datasets. (A–C) The prediction accuracy matrix of PCDS classification and the expression profiles of nine PCDS hub genes in GSE33000, GSE36980, and GSE48350. [file Image1.tif]

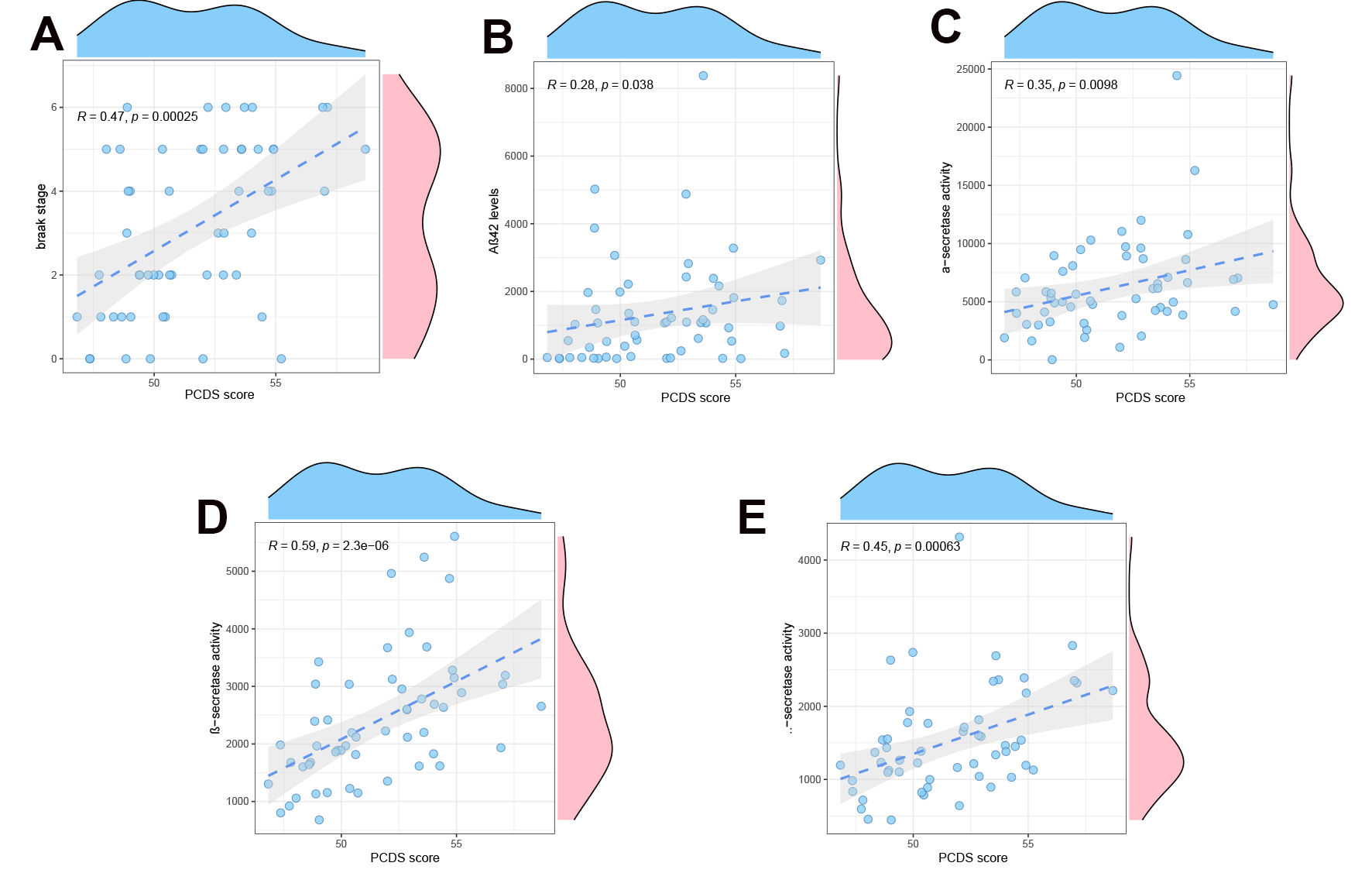

Supplement: Supplementary Figure 2 — The PCDS score predicts the progression of AD. (A–E) The correlation between PCDS score and key AD-related clinicopathological features, including braak stage, Aβ42 levels, α-secretase, β-secretase, and γ-secretase levels. [file Image2.tif]

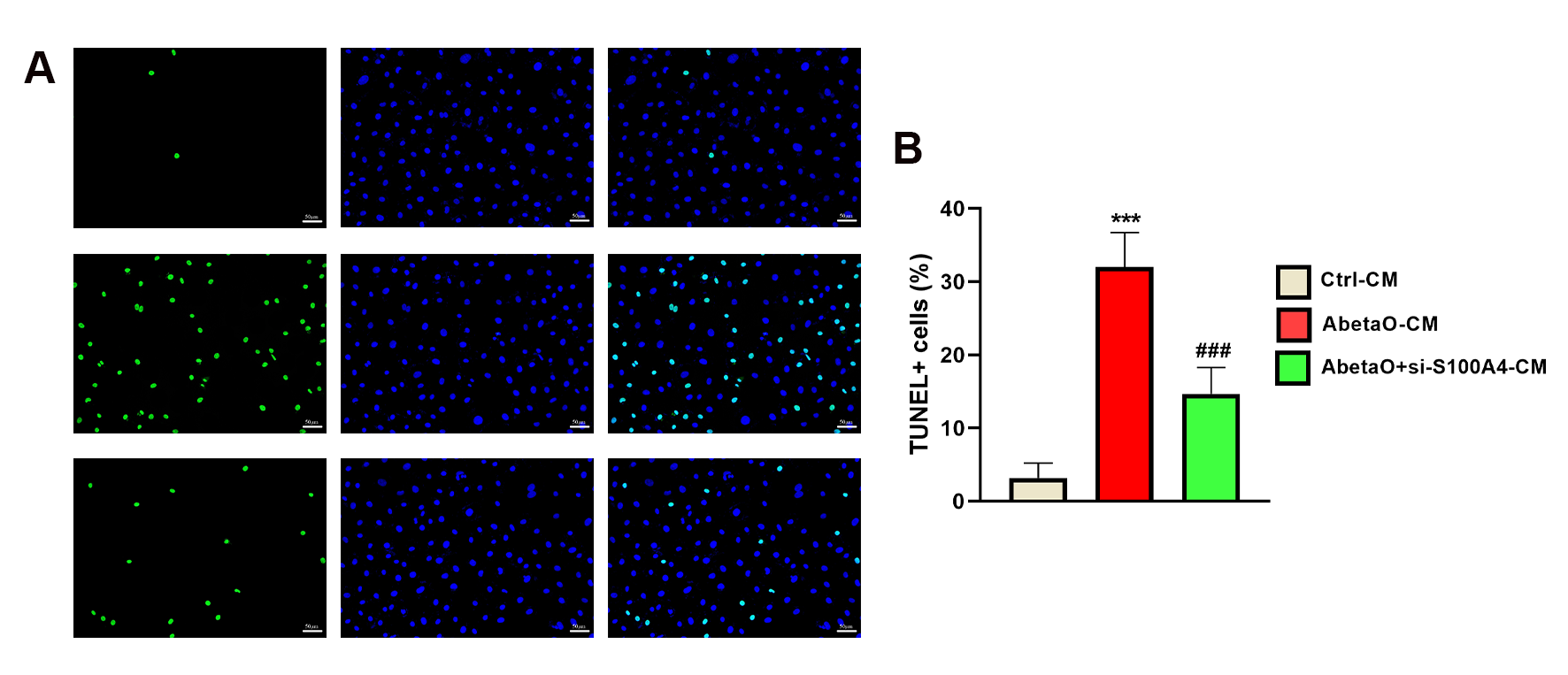

Supplement: Supplementary Figure 3 — Knockdown of S100A4 inhibits neuronal apoptosis. (G–J) TUNEL staining and quantitative results of the cell apoptosis in HT-22 neuronal cells (N=4). Data are presented as mean ± SD. ***p<0.001 vs. Ctrl-CM; ###p< 0.001 vs. AbetaO-CM. [file Image3.tif]
